# Heterozygous variants in the DVL2 interaction region of *DACT1* cause CAKUT and features of Townes–Brocks syndrome 2

**DOI:** 10.1007/s00439-022-02481-6

**Published:** 2022-09-06

**Authors:** Anne Christians, Esra Kesdiren, Imke Hennies, Alejandro Hofmann, Mark-Oliver Trowe, Frank Brand, Helge Martens, Ann Christin Gjerstad, Zoran Gucev, Matthias Zirngibl, Robert Geffers, Tomáš Seeman, Heiko Billing, Anna Bjerre, Velibor Tasic, Andreas Kispert, Benno Ure, Dieter Haffner, Jens Dingemann, Ruthild G. Weber

**Affiliations:** 1 Department of Human Genetics OE 6300, Hannover Medical School, Carl-Neuberg-Str. 1, 30625 Hannover, Germany; 2 Department of Pediatric Kidney, Liver and Metabolic Diseases, Hannover Medical School, Hannover, Germany; 3 Department of Pediatric Surgery, Hannover Medical School, Hannover, Germany; 4 Institute of Molecular Biology, Hannover Medical School, Hannover, Germany; 5 Division of Paediatric and Adolescent Medicine, Oslo University Hospital, Oslo, Norway; 6 Pediatric Nephrology, University Children’s Hospital, Skopje, North Macedonia; 7 Pediatric Nephrology, University Children’s Hospital, Tübingen, Germany; 8 Genome Analytics Research Group, Helmholtz Centre for Infection Research, Brunswick, Germany; 9 Department of Pediatrics, 2nd Faculty of Medicine, Charles University, Prague, Czech Republic

## Abstract

Most patients with congenital anomalies of the kidney and urinary tract (CAKUT) remain genetically unexplained. In search of novel genes associated with CAKUT in humans, we applied whole-exome sequencing in a patient with kidney, anorectal, spinal, and brain anomalies, and identified a rare heterozygous missense variant in the *DACT1* (dishevelled binding antagonist of beta catenin 1) gene encoding a cytoplasmic WNT signaling mediator. Our patient’s features overlapped Townes–Brocks syndrome 2 (TBS2) previously described in a family carrying a *DACT1* nonsense variant as well as those of *Dact1*-deficient mice. Therefore, we assessed the role of *DACT1* in CAKUT pathogenesis. Taken together, very rare (minor allele frequency ≤ 0.0005) non-silent *DACT1* variants were detected in eight of 209 (3.8%) CAKUT families, significantly more frequently than in controls (1.7%). All seven different *DACT1* missense variants, predominantly likely pathogenic and exclusively maternally inherited, were located in the interaction region with DVL2 (dishevelled segment polarity protein 2), and biochemical characterization revealed reduced binding of mutant DACT1 to DVL2. Patients carrying *DACT1* variants presented with kidney agenesis, duplex or (multi)cystic (hypo)dysplastic kidneys with hydronephrosis and TBS2 features. During murine development, *Dact1* was expressed in organs affected by anomalies in patients with *DACT1* variants, including the kidney, anal canal, vertebrae, and brain. In a branching morphogenesis assay, tubule formation was impaired in CRISPR/Cas9-induced *Dact1*^*−/−*^ murine inner medullary collecting duct cells. In summary, we provide evidence that heterozygous hypomorphic *DACT1* variants cause CAKUT and other features of TBS2, including anomalies of the skeleton, brain, distal digestive and genital tract.

**Supplementary Information:**

The online version contains supplementary material available at 10.1007/s00439-022-02481-6.

## Introduction

Congenital anomalies of the kidney and urinary tract (CAKUT) comprise various malformations resulting from defects in the morphogenesis of the kidneys and/or the urinary tract. Kidney anomalies observed within the CAKUT spectrum range from severe manifestations, such as kidney agenesis or multicystic dysplastic kidney (MCDK), to milder phenotypes, including kidney hypoplasia or fused/duplex kidney (Schedl 2007). Taken together, all CAKUT phenotypes have a prevalence of 3–9/1000 live births (Pohl et al. 2002; Queisser-Luft et al. 2002; Stoll et al. 2014), and account for around 40% of cases with end-stage kidney disease in children and adolescents (Harambat et al. 2012). CAKUT occur sporadically in around 85% of patients. In familial cases, inheritance is often dominant. Over 500 syndromes are associated with CAKUT (Limwongse 2009), and around one-third of patients present with extrarenal features (Stoll et al. 2014).

To date, around 60 genes are known to cause isolated or mild syndromic CAKUT in humans if mutated (Kosfeld et al. 2018; van der Ven et al. 2018b). Chromosomal aberrations (Stoll et al. 2014) including microdeletions/-duplications (Weber et al. 2011; Sanna-Cherchi et al. 2018) may also be causative. Despite increasing knowledge about the genetic basis of human CAKUT, the majority of patients remain genetically unexplained (van der Ven et al. 2018a, b). Although identifying new CAKUT-causing genes remains challenging due to high genetic heterogeneity, variable expressivity and incomplete penetrance (van der Ven et al. 2018b), gene discovery has been accelerated by the advent and large-scale use of next generation sequencing (NGS) technologies. Dominant genes associated with human CAKUT using NGS include *DSTYK* (Sanna-Cherchi et al. 2013), *TBX18* (Vivante et al. 2015), *TBC1D1* (Kosfeld et al. 2016), *PBX1* (Heidet et al. 2017), *GREB1L* (Brophy et al. 2017; De Tomasi et al. 2017), *LIFR* (Kosfeld et al. 2017; Christians et al. 2020), *TBX6* (Verbitsky et al. 2019; Yang et al. 2020), *GDF6* (Martens et al. 2020), and *ZMYM2* (Connaughton et al. 2020); recessive genes include *ITGA8* (Humbert et al. 2014) and *ROBO1* (Münch et al. 2022).

NGS techniques have been especially successful in identifying the underlying genetic cause of syndromic CAKUT patients (van der Ven et al. 2018a). In this study, whole-exome sequencing (WES) in a patient presenting with unilateral kidney agenesis and contralateral duplex kidney as well as malformations of the spine, distal digestive tract, and central nervous system yielded a very rare heterozygous variant in the *DACT1* (dapper, dishevelled binding antagonist of beta catenin 1) gene. *DACT1* is a known murine CAKUT gene (Suriben et al. 2009; Wen et al. 2010) encoding a cytoplasmic protein acting in WNT signaling (Cheyette et al. 2002; Zhang et al. 2006). A *DACT1* nonsense variant was described in a family with features overlapping Townes–Brocks syndrome 1 (TBS1, OMIM # 107480) (Webb et al. 2017) referred to as TBS2 (OMIM # 617466). By studying the frequency, clinical impact, and functional consequences of *DACT1* variants in a cohort of CAKUT patients, investigating *Dact1* expression during murine development, and analyzing the consequences of *Dact1* deficiency in an in vitro model of tubulogenesis, we provide further evidence that *Dact1* deficiency and very rare *DACT1* variants may cause kidney and specific extrarenal anomalies in mice and humans.

## Patients and methods

### Patients

The study was approved by the Ethics Boards of Hannover Medical School, Hannover, Germany; Tübingen University Hospital, Tübingen, Germany; Oslo University Hospital, Oslo, Norway; Skopje University Hospital, Skopje, North Macedonia. Each family provided informed consent for participation in the study. Of the 209 CAKUT patients analyzed, 130 were males, 79 were females, and their mean age was 11.5 years (range 2–37 years). All 209 patients had kidney anomalies, 56 were additionally affected by vesicoureteral reflux, and 33 patients had undergone kidney transplantation due to end-stage kidney disease. The spectrum of kidney anomalies with or without urinary tract malformations of the analyzed patients is listed in Supplementary Table 1. Case reports of patients carrying *DACT1* variants are provided in the supplementary material.

### Whole-exome and targeted *DACT1* sequencing

WES was performed on leukocyte DNA of 38 CAKUT patients and 137 individuals not affected by CAKUT (serving as in-house controls for WES data analysis of index patient V005-II.04, Supplementary Table 2) using the SureSelectXT Human All Exon V4 target enrichment kit (Agilent, Santa Clara, CA, USA) on a HiSeq 2000 (Illumina, San Diego, CA, USA) sequencer or the SureSelectXT Human All Exon V5 + UTRs target enrichment kit (Agilent) on a HiSeq 2500 (Illumina) sequencer. All samples were sequenced to a mean coverage of 50x. Sequencing data were aligned to the human reference genome (hg19/GRCh37) using the CLC Genomics Workbench (version 5.0.2; Qiagen, Hilden, Germany). WES data were annotated and prioritized using Ingenuity Variant Analysis (Qiagen) and our in-house NGS data analysis workflow. Supplementary Table 2 summarizes the candidate gene-based strategy used to analyze WES data of index patient V005-II.04. Using conventional chain termination protocols and a 3130XL Genetic Analyzer (Life Technologies, Carlsbad, CA, USA), mutational analysis of all coding exons and adjacent intronic regions of NM_016651.5(*DACT1*) was done to verify variants identified by WES analysis, to determine familial segregation, and to screen for *DACT1* variants in 171 further CAKUT patients. Supplementary Table 3 summarizes the sequences of oligonucleotides used. The minor allele frequencies (MAF) of genetic variants were retrieved from the Genome Aggregation Database (gnomAD controls v2.1.1, total population, https://gnomad.broadinstitute.org/). Variant pathogenicity was predicted using CADD (https://cadd.gs.washington.edu/snv; Kircher et al. 2014; Rentzsch et al. 2019), MutationTaster (http://www.mutationtaster.org/), SIFT (https://sift.bii.a-star.edu.sg/), PROVEAN (http://provean.jcvi.org/index.php), PolyPhen-2 (http://genetics.bwh.harvard.edu/pph2/), and classified using the ACMG guidelines (Richards et al. 2015).

### Animals

All applicable international, national and/or institutional guidelines for the care and use of animals were followed. All experiments were approved by the Ethics Board of the Lower Saxony State Office for Consumer Protection and Food Safety. Murine embryos were derived from matings of Ztm:NMRI wildtype mice. For timed pregnancies, vaginal plugs were checked in the morning after mating, and noon was defined as embryonic day (E) 0.5. Embryos or urogenital systems were dissected in phosphate-buffered saline (PBS) and fixed in 4% paraformaldehyde (PFA) in PBS followed by subsequent dehydration in methanol. Fixed embryos or urogenital systems were stored in 100% methanol at −20 °C prior to RNA in situ hybridization analysis.

### RNA in situ hybridization on sections of murine embryos or kidneys

To determine the expression pattern of *Dact1* during murine embryonic development, non-radioactive RNA in situ hybridization analysis was carried out following a standard protocol (Moorman et al. 2001). In brief, PFA-fixed embryos or urogenital systems of wildtype mice were paraffin-embedded, and sectioned to 10 µm thickness. Sections were deparaffinized in Carl Roth ROTI Histol (#10379029; Thermo Fisher Scientific, Waltham, MA, USA), sequentially rehydrated in ethanol/H_2_O, washed in PBS, and treated with 10 µg/ml proteinase K (#7528; Carl Roth, Karlsruhe, Germany) in 0.1 M Tris, pH 8.0 at 37 °C for 8 min. After washing with 0.2% glycerin/PBS and PBS, and post-fixation with 4% PFA / 0.2% glutaraldehyde at room temperature (RT) for 20 min each, sections were hybridized with a digoxygenin-labeled riboprobe (DIG RNA Labeling Mix, #11277073910; Sigma-Aldrich, St. Louis, MO, USA) directed against mouse *Dact1* mRNA (514 bp, NM_001190466, position 872-1385) in hybridization buffer at 70 °C overnight. Sections were washed twice in 50% formamide / 50% 2xSSC (pH 7.0) at 65 °C for 20 min. Probes were detected using Anti-Digoxigenin-AP, Fab fragments (2 h at RT) and BM-Purple AP substrate (#11093274910 and #11442074001; Sigma-Aldrich). For each developmental stage, at least 3 specimens were analyzed. Stained sections were documented on a Leica DM5000 microscope using a Leica DFC300 FX digital camera (Leica Microsystems, Wetzlar, Germany).

### Cloning of expression constructs and site-directed mutagenesis

To generate a *DACT1* expression construct, the full-length *DACT1* open reading frame was amplified from human cDNA and sub-cloned into the *pcDNA3.1-Myc* vector (Thermo Fisher Scientific) using customized oligonucleotides (Supplementary Table 3) and the In-Fusion HD Cloning Kit (Takara Bio, Kusatsu, Japan). The variants were inserted into the *DACT1* expression construct using customized oligonucleotides (Supplementary Table 3) and the Phusion Site-Directed Mutagenesis Kit (Thermo Fisher Scientific).

### Cell culture and transient transfection

Human embryonic kidney 293T (HEK293T) cells were cultured in high-glucose Dulbecco's Modified Eagle Medium (DMEM; Merck, Darmstadt, Germany) supplemented with 10% fetal bovine serum, 100 units/ml penicillin, and 100 μg/ml streptomycin (all Thermo Fisher Scientific). For murine inner medullary collecting duct 3 (mIMCD3) cells, DMEM/Ham's F-12 (1:1) medium (Merck) was used. Cell cultures were maintained at 37 °C in a humidified atmosphere containing 5% CO_2_. For transient transfection of HEK293T cells, Lipofectamine 3000 transfection reagent (Thermo Fisher Scientific) was used following standard protocols.

### Immunoprecipitation

To analyze binding of wildtype and mutant Myc-DACT1 to Flag-DVL2 (dishevelled segment polarity protein 2) by immunoprecipitation (IP), HEK293T cells (1.0 × 10^7^) were seeded in Petri dishes and transiently co-transfected with *pcDNA3.1-Myc-DACT1* (wildtype or mutant) and *pCMV5-Flag(3x)-DVL2* (#24802; Addgene, Watertown, MA, USA). At 24 h post transfection, cells were lysed in IP buffer (50 mM Tris-HCl, pH 8.0, 50 mM sodium fluoride, 1 mM sodium orthovanadate, 1% Triton X-100) supplemented with protease and phosphatase inhibitors (Roche Diagnostics, Mannheim, Germany). After adding 1 µg of anti-Myc antibody (#sc-40; Santa Cruz Biotechnology, Dallas, TX, USA), lysates were rotated overnight at 4 °C. Protein G Sepharose beads (GE, Boston, MA, USA) were equilibrated in IP buffer and incubated with the lysates for 4 h at 4 °C. After washing 5 × with IP lysis buffer, proteins eluted from the beads using Laemmli buffer (62.5 mM Tris-HCl, pH 6.8, 10% glycerin, 2% sodium dodecyl sulfate (SDS), 5% 2-mercaptoethanol, 1 mM ethylenediaminetetraacetic acid, 0.01% bromophenol blue) were detected by Western blot analysis.

### Western blot analysis

After SDS-polyacrylamide gel electrophoresis and semi-dry electro-blotting, nitrocellulose membranes (GE) were treated with 5% fat-free milk powder dissolved in PBS with 0.05% Tween 20 (PBST) as blocking agent. The primary antibodies mouse anti-Myc (#sc-40; Santa Cruz Biotechnology) or mouse anti-Flag (#8146; Cell Signaling Technology, Danvers, MA, USA) were diluted at 1:1,000 in 5% (w/v) bovine serum albumin in PBST and used for immunodetection. After incubation overnight at 4 °C, membranes were washed with PBST, exposed to the secondary horseradish peroxidase-conjugated anti-mouse antibody (#sc-2962; Santa Cruz Biotechnology; dilution 1:3,000) in 5% fat-free milk powder dissolved in PBST for 90 min at RT, washed again with PBST, and developed using the SuperSignal West Dura Extended Duration Substrate (Thermo Fisher Scientific). Signals were acquired using the Fusion FX7 gel documentation system (Vilber, Collégien, France). Densitometric quantification of protein bands was performed using ImageJ software (Schneider et al. 2012).

### Qualitative *DACT1* mRNA expression analysis

To determine whether mIMCD3 cells express *Dact1*, RNA was isolated from mIMCD3, HEK293T (positive control), and HeLa (negative control) cells (estimation of RNA expression levels based on www.proteinatlas.org) using the RNeasy Mini Kit (Qiagen). cDNA was synthesized from RNA samples using the Superscript IV First-Strand Synthesis System (Thermo Fisher Scientific). Exon-spanning oligonucleotides specific for murine *Dact1* cDNA (NM_021532.4, c.390-518) or human *DACT1* cDNA (NM_016651.6, c.487-709) were used for PCR amplification (Supplementary Table 3), respectively. Sequencing of the generated amplicons was done using conventional chain termination protocols, as described above.

### CRISPR/Cas9 genomic engineering

To generate a *Dact1* knockout cell model, mIMCD3 cells and a protocol for CRISPR/Cas9-mediated RNA-guided genome editing (Ran et al. 2013) were used. In brief, a single guide RNA (sgRNA) targeting the first exon of *Dact1* (targeted sequence 5’-GCG TAC CCG CGA GCG CCA GG-3’) was designed using the CRISPOR web-based tool (http://crispor.tefor.net), and sense and antisense oligonucleotides (Supplementary Table 3) were synthesized (Eurofins Genomics, Ebersberg, Germany). The dimerized oligonucleotides were inserted into a *Bpi*I-digested *pSpCas9(BB)-2A-GFP* plasmid (#48138; Addgene), containing a sgRNA scaffold and expression cassettes for Cas9 and GFP. By transient transfection, the resulting construct was introduced into mIMCD3 cells. GFP-positive cells were isolated 24 h after transfection at the Cell Sorting Core Facility of Hannover Medical School using a MoFlo XDP cell sorter (Beckman-Coulter, Brea, MA, USA). To identify the genotype of selected cell clones, their DNA was extracted using the innuPREP DNA Mini Kit (Analytik Jena, Jena, Germany), and PCR products of *Dact1* exon 1 were analyzed by direct sequencing (oligonucleotides listed in Supplementary Table 3). For allele-specific sequence analysis, the PCR product was cloned into a *pcDNA3.1* vector (Invitrogen, Carlsbad, CA, USA) using oligonucleotides listed in Supplementary Table 3, and the plasmid DNA of at least 10 *Escherichia coli* transformants were analyzed by direct sequencing. In the three mIMCD3 cell clones selected for further analysis harboring either *Dact1* wildtype (*Dact1*^+/+^; clone 2) or a biallelic knockout (*Dact1*^−/−^; clones 11 and 12), all 13 coding off-target sites were analyzed by direct sequencing (oligonucleotides given in Supplementary Table 3) to ascertain absence of mutation.

### Tubulomorphogenesis assay

To investigate the consequences of a knockout of *Dact1* on branching morphogenesis, a tubulomorphogenesis assay was performed using mIMCD3 cells, as previously described (De Tomasi et al. 2017). In brief, mIMCD3 cells were cultured in a three-dimensional (3-D) gel of collagen type I from rat tail (Corning, Corning, NY, USA) in 12-well plates. For each experiment, each cell clone was plated in duplicate. A thin collagen layer without cells was applied to the well, followed by a collagen layer containing 100,000 cells/ml. After the gel had solidified, 500 µl of DMEM/Ham's F-12 (1:1) medium (Merck) supplemented with 10% fetal bovine serum, 100 units/ml penicillin, and 100 µg/ml streptomycin (all purchased from Thermo Fisher Scientific) were added to the well. After seven days of cultivation, cells were documented using an inverted microscope (DM IL LED Fluo, Leica Microsystems) equipped with an EC3 camera (Leica Microsystems). For better visualization, 3-D cultures were fixed in 4% PFA in PBS and stained with Alexa Fluor 488 Phalloidin (#A12379; Invitrogen; dilution 1:200 in PBST). For quantification, images of 3-D cultures were blinded, and at least 60 cellular structures were classified as tubular or spherical for each cell clone in each experiment (*n* = 3).

### Statistical analysis

Statistical significance was calculated using Student’s *t* test or Fisher’s exact test (two-tailed), whereby *p* values of ≤ 0.05 were considered significant, and *p* values of ≤ 0.01 highly significant.

## Results

### Very rare heterozygous *DACT1* variants predicted to be deleterious were identified in eight of 209 (3.8%) families with kidney anomalies

**Fig. 1:**
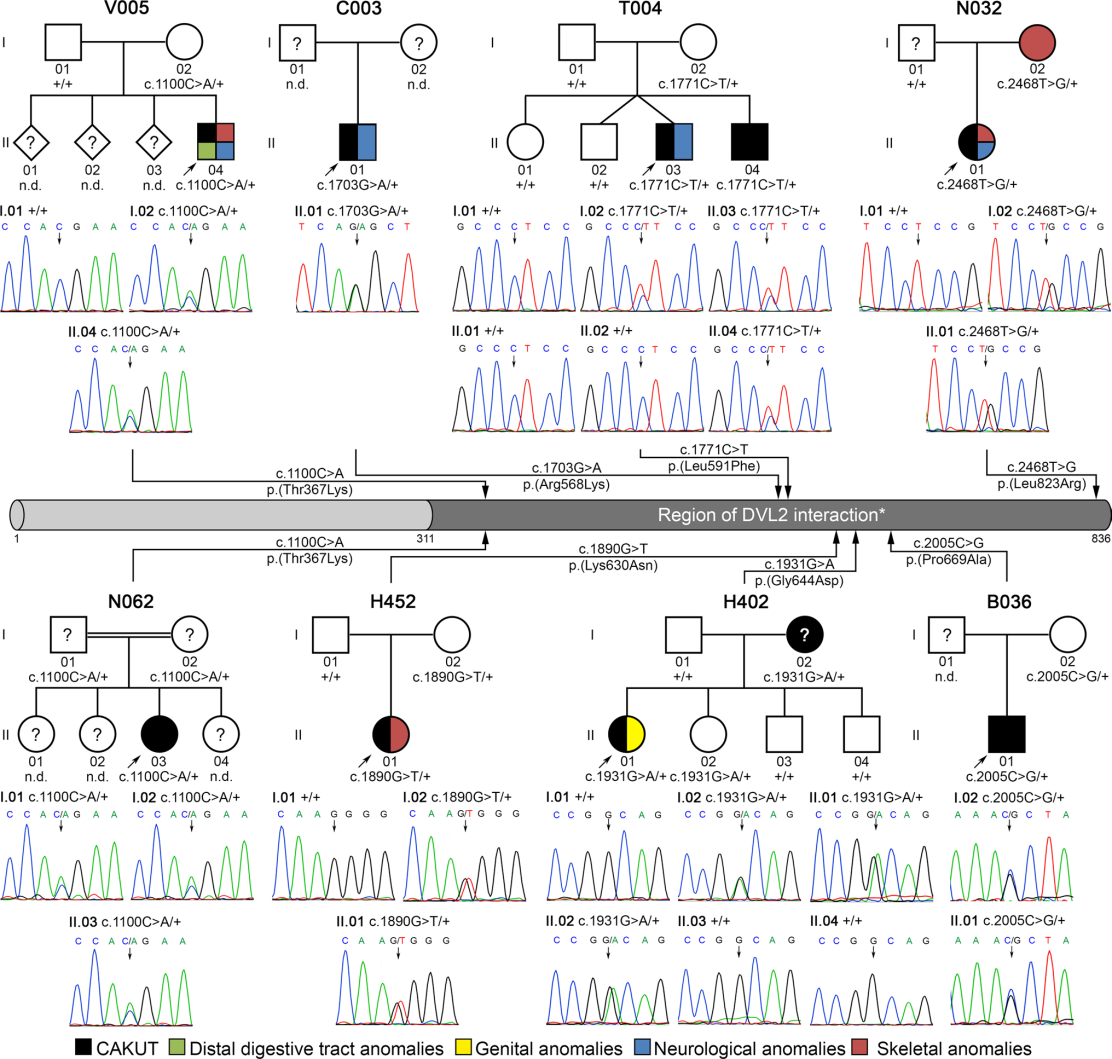
Identification of very rare heterozygous *DACT1* missense variants in eight of 209 CAKUT families (3.8%). Electropherograms of *DACT1* variants (affected base positions are indicated by arrows), their segregation, and the localization of affected amino acid residues within a representation of the DACT1 protein are shown. All seven different variants are located within the region of DVL2 interaction (*) according to Zhang et al. (2006). In pedigrees, squares denote males, circles females, and colored symbols affected individuals with phenotypes as indicated. Six of eight (75%) index CAKUT patients (indicated in pedigrees by an arrow) that received reverse phenotyping presented with extrarenal features including distal digestive tract anomalies, skeletal, genital and/or neurological anomalies. Black question marks denote family members with no clinical information available or without kidney ultrasound. Black circle with white question mark denotes individual with putative left-sided non-obstructive duplex kidney. Individual V005-II.04 was analyzed by whole-exome sequencing. Validation of the variant, identification of variants in further patients, and segregation analysis were performed by direct sequencing. + , represents *DACT1* wildtype sequence; n.d., individual with non-available DNA

**Fig. 2:**
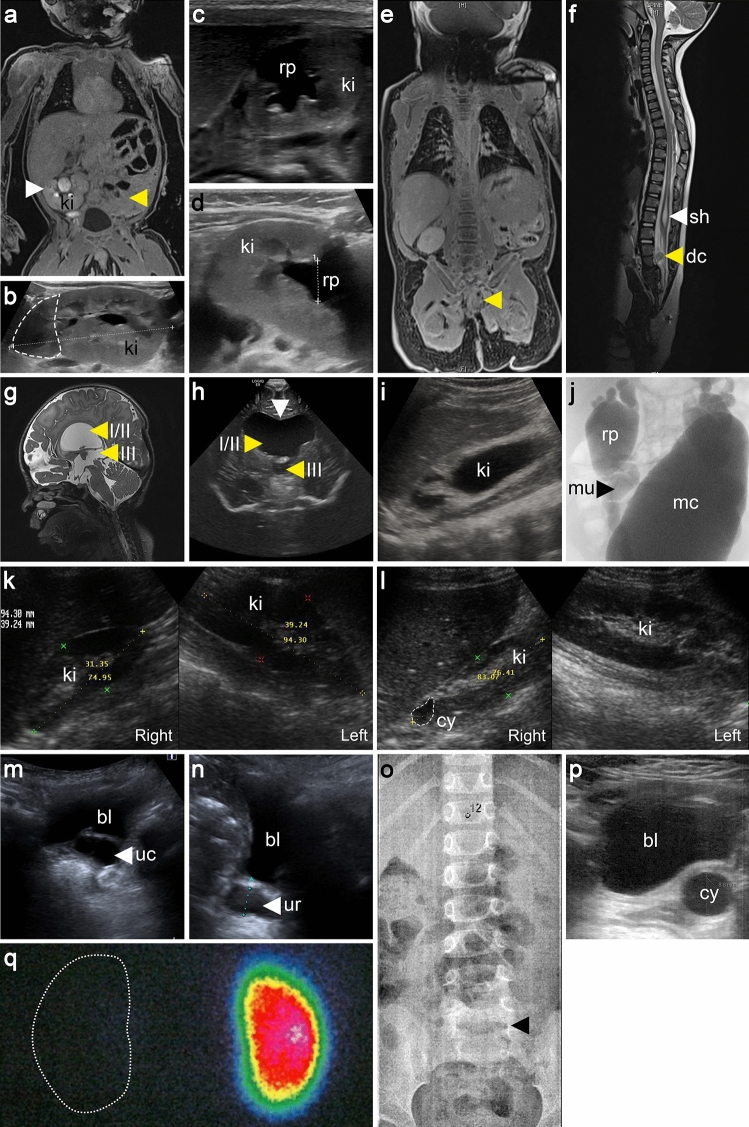
Phenotype spectrum of patients with *DACT1* variants. **a**–**h** The index patient, V005-II.04, presented with numerous anomalies, including left-sided kidney agenesis (yellow arrow) and right-sided malrotated duplex kidney (ki) (white arrow) on magnetic resonance imaging (MRI) scan (**a**). By kidney ultrasound (US), the duplex kidney (marked by a dashed line, **b**) shows a dilated kidney pelvis (rp, **c**, **d**). By MRI scan, a caudal regression syndrome with missing coccyx and sacral dysplasia (yellow arrow, **e**), syringohydromyelia (sh) at T11-T12 (white arrow, **f**), and an intraspinal dermoid cyst (dc) at L2-L3 (yellow arrow, **f**) were diagnosed, along with a triventricular hydrocephalus internus (ventricles I-III, yellow arrows, **g, h**) due to an aqueductal stenosis, and an agenesis of the septum pellucidum (white arrow, **h**) in the central nervous system. **i, j** Patient T004-II.03 presented with bilateral kidney dysplasia on kidney US (right-sided kidney with hydronephrosis shown, **i**), and dilated kidney pelvises, megaureters (mu), and megacystis (mc) upon micturating cysto-urethrogram (**j**). **k** By kidney US of patient H452-II.01, a right-sided kidney hypoplasia and a left-sided normal kidney were diagnosed. **l** Patient H402-II.01 presented with right-sided kidney hypoplasia with a cyst (cy) and normal left-sided kidney on kidney US. **m, n** Kidney US of patient B036-II.01 showed a left-sided dilated residual ureter (ur) ending in a ureterocele (uc) located in the bladder (bl). **o**–**q** Patient N032-II.01 presented with a lumbarization of S1 (black arrow, **o**) on spinal X-ray (T12 is marked), no radiotracer uptake equivalent to a missing functional kidney on the left due to a multicystic dysplastic kidney (residues of which are seen as a cystic structure next to the bladder on US, **p**) and normal uptake in the right kidney on dimercaptosuccinic acid (DMSA) kidney scan (**q**)

Under the assumption that NGS techniques are especially successful in identifying the genetic cause in syndromic CAKUT patients, we performed WES on leukocyte DNA of a four-year-old male index patient, V005-II.04, the child of non-consanguineous Kurdish parents (Fig. 1), born with a caudal regression syndrome including kidney, anorectal, and spinal anomalies and brain malformations (Fig. 2, Table 1). Kidney ultrasound, magnetic resonance imaging, and isotope nephrography were notable for left-sided kidney agenesis and a right-sided malrotated duplex kidney with hydronephrosis and primary obstructive megaureter (Fig. 2a–d). Additionally, a caudal regression syndrome with missing coccyx, sacral dysplasia, syringohydromyelia, an intraspinal dermoid cyst, anorectal agenesis with rectourethral fistula, and neurogenic bladder were diagnosed (Fig. 2e, f). In the central nervous system, agenesis of the septum pellucidum, triventricular hydrocephalus internus due to aqueductal stenosis, and agenesis of the cerebellar vermis were noted (Fig. 2g, h). Further details are provided in the supplementary material. WES data were analyzed using a candidate gene-based strategy and our in-house NGS data analysis pipeline. By prioritizing good-quality, non-silent, and very rare (MAF ≤ 0.0005) variants not present in in-house control individuals and known or presumed to cause isolated or syndromic CAKUT in humans or mice (Supplementary Table 2), we identified a very rare (MAF = 0.000333 according to gnomAD controls) missense variant in the *DACT1* gene, NM_016651.5(*DACT1*):c.1100C > A p.(Thr367Lys). This variant is predicted to be deleterious by SIFT, PROVEAN, and PolyPhen-2, and has a CADD score of close to 20 (19.40) predicting that it is among the top 1.15% most deleterious variants in the human genome. The variant was confirmed to be heterozygous and shown to be inherited from the patient’s unaffected mother by targeted sequencing (Fig. 1). No other heterozygous or biallelic variants of interest, especially none in known or presumed CAKUT-associated genes in humans or mice, were identified in the patient. Considering that a family with a *DACT1* nonsense variant (Webb et al. 2017) and *Dact1* knockout mice (Suriben et al. 2009; Wen et al. 2010) had similar phenotypes involving the urogenital system, distal digestive tract, and spine, the *DACT1* variant was considered to be causative and to explain the phenotype of our patient.

**Table 1:** Very rare (MAF ≤ 0.0005) heterozygous *DACT1* variants identified in eight of 209 CAKUT families (3.8%)

Case (gender, year of birth, country of origin)	Chr. position^a^/ dbSNP ID	Nucleotide alteration^a^	Deduced protein change^a^	MAF^b^	Pathogenicity prediction^c^	Inheritance	Anomalies of the kidneys and urinary tract^d^	Extrarenal anomalies^d^	Comparison of variant frequency in CAKUT cases vs. controls^e^	Clinical interpretation according to ACMG guidelines^f^
V005-II.04 (male, *2016, Kurdish)	14:59,112,441/rs564164674	c.1100C > A	p.(Thr367Lys)	0.000333	19.40/ polymorphism/ damaging/ deleterious/ possibly damaging	Maternal	Kidney agenesis (l); malrotated duplex kidney, hydronephrosis, primary obstructive megaureter (r), neurogenic bladder	Caudal regression syndrome with missing coccyx, sacral dysplasia, syringohydromyelia, intraspinal dermoid cyst, anorectal agenesis with rectourethral fistula, agenesis of the septum pellucidum, triventricular hydrocephalus internus due to an aqueductal stenosis, agenesis of the cerebellar vermis	*p* = 0.0094	Likely pathogenic (PS3, PS4, PP3)
N062-II.03 (female, *2004, Turkey)	14:59,112,441/rs564164674	c.1100C > A	p.(Thr367Lys)	0.000333	19.40/ polymorphism/ damaging/ deleterious/ possibly damaging	Both parents are carriers	MCDK (r)	None	*p* = 0.0094	Likely pathogenic (PS3, PS4, PP3)
C003-II.01 (male, *2000, Czech Republic)	14:59,113,044/ –	c.1703G > A	p.(Arg568Lys)	–	11.68/ polymorphism/ tolerated/ neutral/ benign	n.d.	Cystic kidney dysplasia (bilat)	Intrauterine growth retardation, mental retardation, autism, epilepsy	*p* = 0.0035	Uncertain significance (PS3, PS4, BP4)
T004-II.03 (male, *2008, Greece)	14:59,113,112/rs369268433	c.1771C > T	p.(Leu591Phe)	0.000048	14.95/ polymorphism/ damaging/ neutral/ possibly damaging	Maternal	Kidney dysplasia, hydronephrosis, megaureter, VUR (all bilat), megacystis	Short stature, learning disability (overall IQ of 71)	*p* = 0.0238	Likely pathogenic (PS3, PS4, PP3)
H452-II.01 (female, *2006, North Macedonia)	14:59,113,231/rs201251394	c.1890G > T	p.(Lys630Asn)	0.000354	14.86/ polymorphism/ damaging/ neutral/ possibly damaging	Maternal	Kidney hypoplasia (r)	High arched palate	*p* = 0.1414	Uncertain significance (PS3, PP3)
H402-II.01 (female, *2002, North Macedonia)	14:59,113,272/ rs754474567	c.1931G > A	p.(Gly644Asp)	0.000085	17.10/ disease causing/ tolerated/ neutral/ benign	Maternal	Kidney hypoplasia with a cyst, VUR (all r)	Dextroposition of the uterus, ovarian cyst (r)	*p* = 0.0396	Uncertain significance (PS3, PS4, BP4)
B036-II.01 (male, *2015, Germany)	14:59,113,346/ –	c.2005C > G	p.(Pro669Ala)	–	25.00/ disease causing/ damaging/ deleterious/probably damaging	Maternal	MCDK, dilated residual ureter ending in ureterocele (all l)	None	*p* = 0.0035	Likely pathogenic (PS3, PS4, PP3)
N032-II.01 (female, *2014, Afro-American)	14:59,113,809/ –	c.2468T > G	p.(Leu823Arg)	–	24.70/ disease causing/ damaging/ deleterious/probably damaging	Maternal	MCDK (l)	Lumbarization of S1, high arched palate, narrow forehead, bone protrusion between the eyes, flattened/concave temporal bones, epicanthus, delayed psychomotor development, autism (atypical)	*p* = 0.0035	Likely pathogenic (PS3, PS4, PP3)

^a^Reference sequence: NM_016651.5, genome build: GRCh37/hg19

^b^According to gnomAD controls v2.1.1, total population

^c^Pathogenicity prediction according to CADD: ≥ 15 (rounded) considered pathogenic, ≥ 20 predicted to be among the top 1% most deleterious variants in the human genome (Kircher et al. 2014; Rentzsch et al. 2019) / MutationTaster / SIFT / PROVEAN / PolyPhen-2

^d^Selected phenotypes are shown in Fig. 2, findings in variant carriers of this study and the literature are summarized in Supplementary Table 4, case reports are provided in supplementary material

^e^The variant frequency in our cohort (*n* = 209) was compared to gnomAD controls v2.1.1, total population; *p *values were calculated using the two-tailed Fisher’s exact test

^f^According to Richards et al. (2015), also considering biochemical variant characterization (Fig. 4c, d), whereby PS3 was rated as moderate

*bilat* bilateral, *l* left, *MAF* minor allele frequency, *MCDK* multicystic dysplastic kidney, *n.d.* not determined, *r* right, *VUR* vesicoureteral reflux, *-* not available

To determine the frequency of *DACT1* variants in a cohort of CAKUT patients, 208 additional families with kidney malformations (Supplementary Table 1) were subjected to WES or targeted *DACT1* sequencing. We identified very rare (MAF ≤ 0.0005 according to gnomAD controls) missense variants in seven further families (Fig. 1). The variant carried by the index patient, c.1100C > A p.(Thr367Lys), was detected in a second patient (Fig. 1, Table 1). Two variants, c.2005C > G p.(Pro669Ala) and c.2468T > G p.(Leu823Arg), are not listed in the gnomAD database and have a CADD score ≥ 20, indicating that they are considered to be among the top 1% most deleterious variants in the human genome (Table 1). All variants except c.1703G > A p.(Arg568Lys) were deleterious according to at least two of five prediction tools (i.e., CADD, MutationTaster, SIFT, PROVEAN, PolyPhen2). Four variants were classified as likely pathogenic according to the ACMG guidelines, while three variants were of uncertain significance (Table 1). Each variant except c.1890G > T p.(Lys630Asn) was significantly more frequent in our cohort of CAKUT families compared to gnomAD controls (Table 1). Taken together, very rare (MAF ≤ 0.0005) non-silent *DACT1* variants were found in eight of 209 (3.8%) families with kidney anomalies compared to 1,006 of 60,146 (1.7%) individuals from the gnomAD control cohort. This difference is statistically significant (*p* = 0.03, two-tailed Fisher’s exact test). Moreover, in three families we observed co-segregation of *DACT1* variants with CAKUT or extrarenal phenotypes, i.e., megacystis in T004-II.03 and T004-II.04, kidney anomalies in H402-I.02 and H402-II.01, and skeletal anomalies in N032-I.02 and N032-II.01 (not all family members were available for genetic testing or phenotypic evaluation; Fig. 1, Supplementary Table 4). The *DACT1* variants were either maternally inherited (6/8 families) or inheritance could not be determined (2/8 families; Fig. 1).

### *DACT1* variants convey a characteristic phenotype consisting of kidney plus anorectal, genital, skeletal or neurological anomalies in three quarters of CAKUT patients carrying *DACT1* variants

In a reverse phenotyping effort, clinical or radiological reevaluation was performed of the CAKUT index patients from the eight families carrying very rare non-silent *DACT1* variants. All eight patients presented with kidney phenotypes, i.e., unilateral kidney agenesis and contralateral malrotated duplex kidney with hydronephrosis (1/8 patients), unilateral multicystic dysplastic kidney (MCDK) (3/8), bilateral kidney dysplasia with or without cysts (2/8), or unilateral kidney hypoplasia with or without cysts (2/8) (Table 1, Fig. 2). Four patients were additionally diagnosed with anomalies of the urinary tract, i.e., megaureter (1/8), megaureter, vesicoureteral reflux (VUR) and megacystis (1/8), blind ending ureter (1/8), or VUR (1/8) (Table 1, Fig. 2). Notably, six of the eight (75%) CAKUT patients harboring *DACT1* variants also presented with extrarenal anomalies similar to those described in a family with a heterozygous *DACT1* loss-of-function variant (Webb et al. 2017) and in *Dact1*-deficient mice (Suriben et al. 2009; Wen et al. 2010). These include anomalies of the distal digestive tract, e.g., anorectal agenesis with recto-urethral fistula (1/8), genital features, e.g., anomalies of the uterus and ovary (1/8), skeletal features, e.g., spinal and craniofacial anomalies (3/8), and/or neurological features, e.g., malformations of the central nervous system, intellectual disability, autism (4/8) (Table 1, Fig. 2). Further details are provided in the supplementary material. Conversely, gastrointestinal, genital, skeletal or neurological anomalies were only detected in 64 of the 198 (32%) CAKUT patients without very rare non-silent *DACT1* variants of whom information was available. Therefore, CAKUT patients with versus without very rare non-silent *DACT1* variants were significantly more likely to present with extrarenal features in the digestive or genital tracts, skeleton or central nervous system (6/8, 75% versus 64/198, 32%; *p* = 0.02, two-tailed Fisher’s exact test).

### *Dact1* expression during early murine development was detected in organs showing defects in *Dact1*-deficient mice and patients carrying *DACT1* variants

**Fig. 3:**
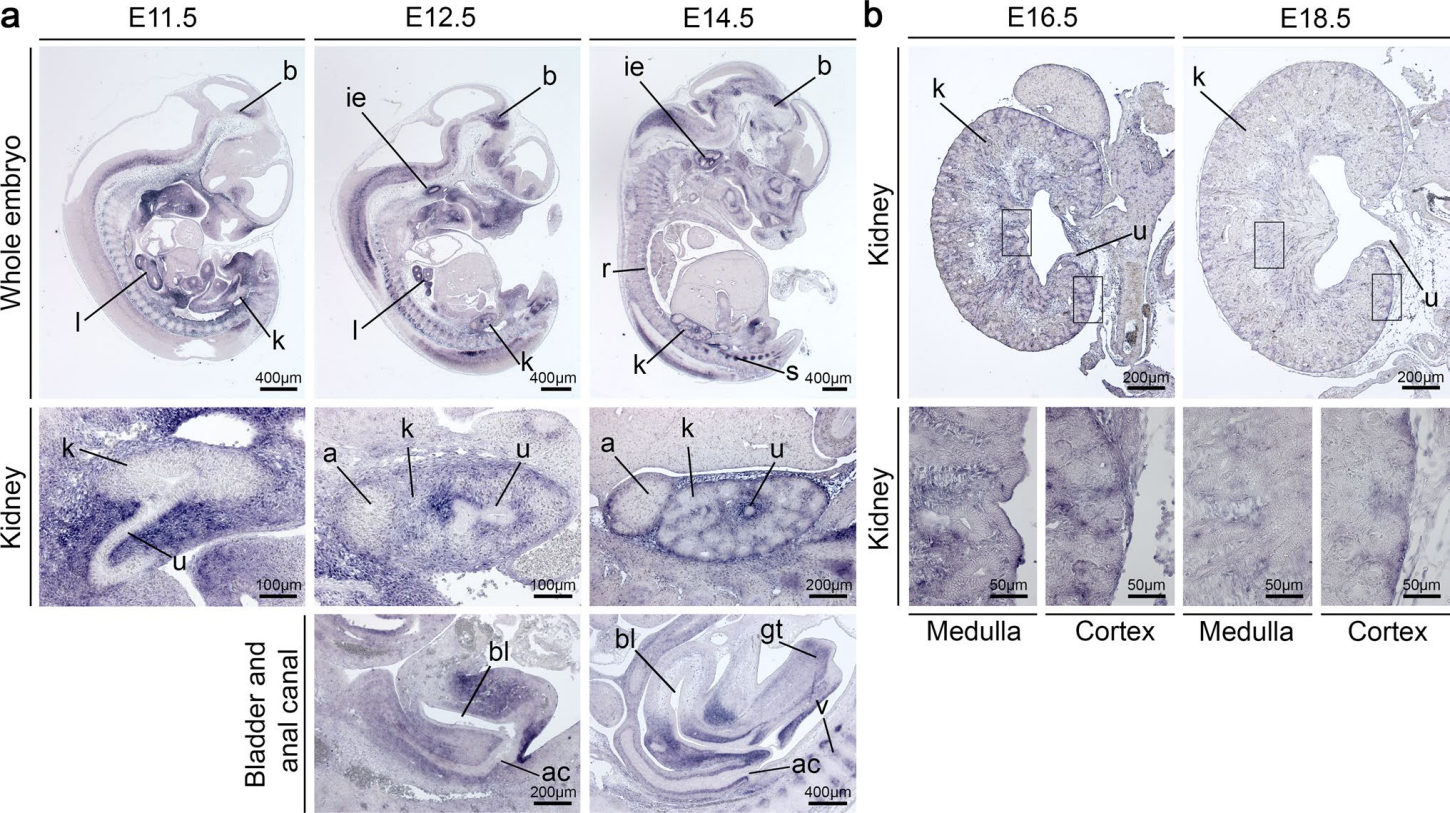
*Dact1* expression pattern in murine embryonic development by non-radioactive RNA in situ hybridization. **a** On sections of murine embryos at E11.5, E12.5 and E14.5, *Dact1* mRNA was detected in a variety of organs, including the kidney, bladder, anal canal, genital tubercle, lung, inner ear, brain, spinal ganglia, vertebrae, and ribs. Expression in the upper urinary tract was found in the mesenchyme of the ureter at E11.5, but not in the metanephric mesenchyme. At E12.5 and E14.5, *Dact1* was additionally expressed in the capsular, cortical, and medullary stroma of the kidney. Similarly, in bladder, urethra, and anal canal *Dact1* expression was confined to mesenchymal cells. **b** On kidney sections at E16.5 and E18.5, *Dact1* expression, which starts to be reduced at E16.5 and is strongly diminished at E18.5, was detected in the mesenchyme of the ureter, the kidney capsule, and the stroma of medulla and cortex (higher magnification images). a, adrenal gland; ac, anal canal; b, brain; bl, bladder; gt, genital tubercle; ie, inner ear; k, kidney; l, lung; r, ribs; s, spinal ganglia; u, ureter; v, vertebrae. For each embryonic stage, at least 3 specimens were analyzed. Scale bars are as indicated

*Dact1-*deficient mice (Suriben et al. 2009; Wen et al. 2010) and patients carrying *DACT1* variants (Webb et al. 2017) (Fig. 2, Table 1) show developmental defects that belong to the caudal regression syndrome including caudal vertebrae agenesis, anal atresia, kidney malformations, aberrantly ending ureters, bladder agenesis, and genital anomalies. In some patients carrying *DACT1* variants, malformations of the brain and/or intellectual disability were also observed (Webb et al. 2017) (Fig. 2, Table 1). Detailed analysis of *Dact1* expression in affected structures, especially in the kidney, during development has not been performed previously. Therefore, we analyzed the spatial and temporal expression of *Dact1* in wildtype mouse embryos using RNA in situ hybridization on whole-embryo sections at E11.5, E12.5, and E14.5 (Fig. 3a), and on kidney sections at E16.5 and E18.5 (Fig. 3b). We detected *Dact1* expression in organs of the caudal region including vertebrae, anal canal, kidney, bladder, genital tubercle as well as in the brain, spinal ganglia, inner ear, lung, and ribs (Fig. 3a). Expression in the upper urinary tract was confined to the mesenchyme of the ureter, and to the capsular, cortical, and medullary stroma of the kidney. Expression at these sites strongly decreased after E14.5 (Fig. 3a, b). *Dact1* expression during early murine development is, therefore, observed in organs showing anomalies in *Dact1*-deficient mice and patients carrying *DACT1* variants.

### *Dact1* knockout impairs tubule formation in a cellular model of branching morphogenesis

**Fig. 4:**
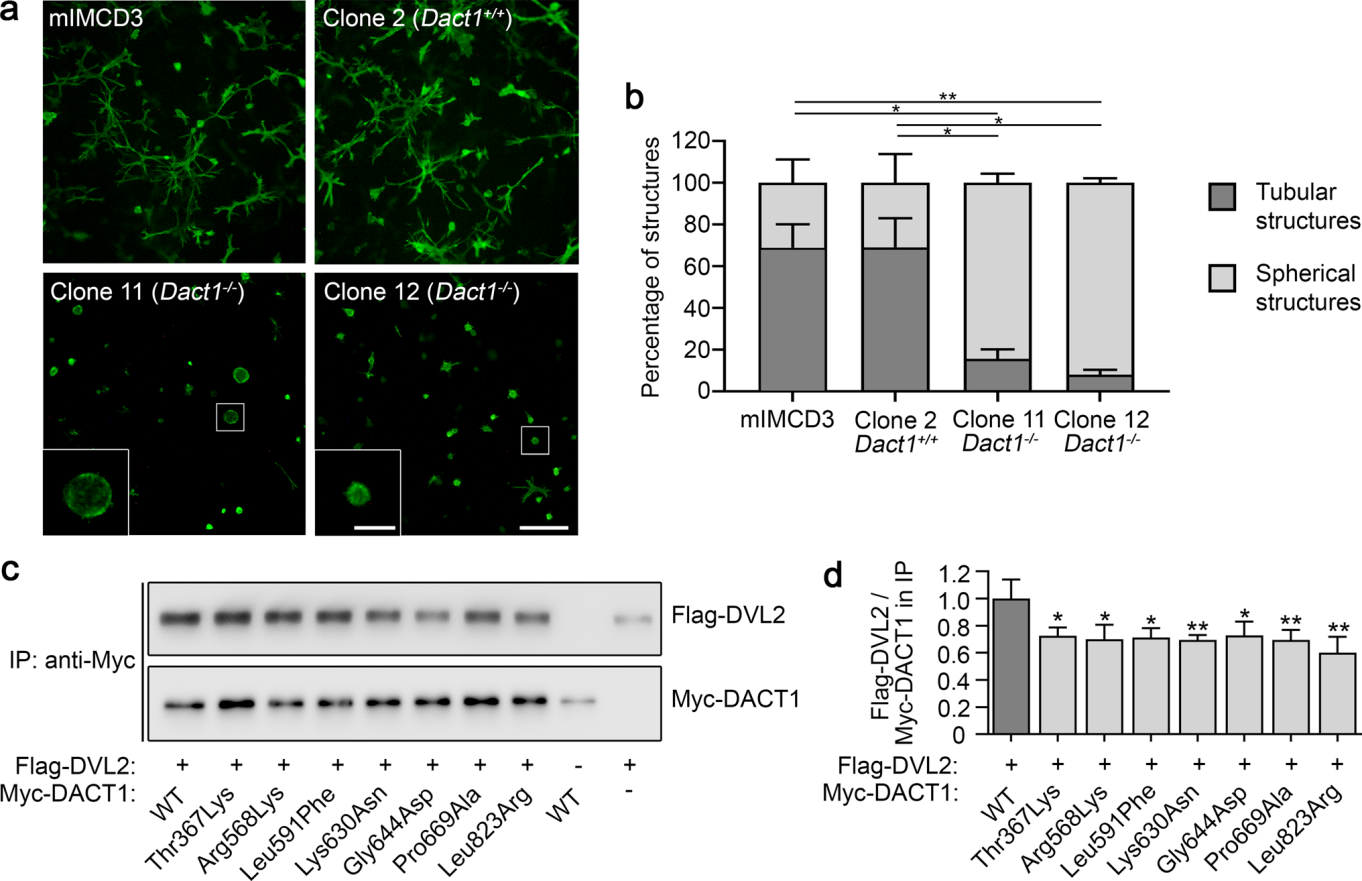
Characterization of *Dact1*-deficient mIMCD3 cells and DACT1 mutant proteins. **a**, **b** To analyze the relevance of *DACT1* for tubulogenesis, a process of major relevance for kidney development, Dact1^*−/−*^ mIMCD3 cells (clones 11 and 12) and control cells (clone 2, *Dact1*^+*/*+^), generated by CRISPR/Cas9 technology (Supplementary Fig. 2), were cultured in a 3-D collagen I matrix for seven days and stained with Alexa Fluor 488 phalloidin (scale bar represents 200 µm) (**a**). Quantification of the tubulomorphogenesis assay showed that more than 70% of mIMCD3 and clone 2 (*Dact1*^+*/*+^) cells developed tubular structures, whereas both *Dact1*^*−/−*^ cell lines (clone 11 and clone 12) displayed nearly no tubuli. At least 60 structures were counted and rated as tubular or spherical for each cell line in each experiment (mean ± SD of three independent experiments) (**b**). **c**, **d** To explore the pathogenicity of the identified *DACT1* variants, interaction of mutant DACT1 with DVL2 was analyzed by co-immunoprecipitation (IP) (**c**). The ratio of Flag-DVL2 to wildtype or mutant Myc-DACT1 in the immunoprecipitates was significantly decreased for all mutants compared to wildtype DACT1 (mean ± SD of four independent experiments) (**d**) indicating impaired DVL2 binding of the DACT1 mutants detected here. *p ≤ 0.05; **p ≤ 0.01 (Student’s *t* test)

To characterize the impact of DACT1 on a process of major relevance for kidney development, we analyzed its role in a 3-D tubulomorphogenesis assay using mIMCD3 cells, a cellular model of branching morphogenesis (Chen et al. 2004; Mai et al. 2005; De Tomasi et al. 2017). Murine IMCD3 cells undergo tubulogenesis in a 3-D collagen gel (Chen et al. 2004), a process disrupted after knockout of relevant genes, such as *GREB1L* (De Tomasi et al. 2017). Here, in mIMCD3 cells, shown to express *Dact1* by RT-PCR (Supplementary Fig. 1), a *Dact1* knockout cell line was generated using CRISPR/Cas9 technology. For subsequent analysis, we selected a wildtype clone without mutational event at the sgRNA on-target site (clone 2, *Dact1*^+*/*+^), and two knockout clones with different biallelic *Dact1* frameshift variants (clones 11 and 12, *Dact1*^*−/−*^) predicted to result in truncated non-functional proteins (Supplementary Fig. 2). As depicted in Fig. 4a and b, over 70% of unmodified mIMCD3 cells and clone 2 (*Dact1*^+*/*+^) control cells formed elongated tubular structures after seven days in a 3-D collagen gel, whereas both *Dact1*^*−/−*^ cell clones failed to form tubules and grew as spherical structures. These data provide evidence that DACT1 is involved in tubulogenesis in vitro.

### Binding of DVL2 to DACT1 mutants is reduced

Human DACT1 interacts with DVL2, a WNT signaling mediator, inducing DVL2 degradation and antagonizing WNT signaling (Zhang et al. 2006). This interaction is mediated by central and C-terminal domains of DACT1, i.e., approximately amino acids 311–836 in the human protein (Zhang et al. 2006; Suriben et al. 2009). Remarkably, 8 of 8 (100%) CAKUT families carry very rare non-silent *DACT1* variants that affect amino acids located within the putative DVL2 interaction region of DACT1 (Fig. 1), while this is only the case in 833 of 1,006 (83%) gnomAD controls carrying very rare non-silent *DACT1* variants (listed in Supplementary Table 5). To explore the functional consequence of the seven different *DACT1* missense variants detected here, we determined DVL2 binding of DACT1 wildtype and mutant proteins in a co-immunoprecipitation assay (Fig. 4c). All seven mutant proteins showed significantly impaired DVL2 binding compared to wildtype DACT1 (Fig. 4d), suggesting that the identified variants act as hypomorphs that may fail to regulate WNT signaling.

## Discussion

**Fig. 5:**
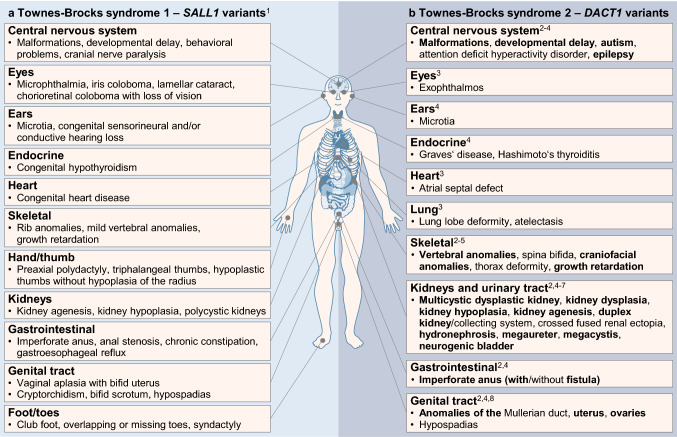
Comparison of Townes–Brocks syndrome 1 and 2 according to the literature and this study. **a**, **b** Schematic representation of the most prominent phenotypical features of TBS1 caused by heterozygous variants in the *SALL1* gene (Kohlhase et al. 1998; Kohlhase 2007) (**a**) and of TBS2 caused by heterozygous variants in the *DACT1* gene (Shi et al. 2012; Nicolaou et al. 2016; Xing et al. 2016; Heidet et al. 2017; Webb et al. 2017; Connaughton et al. 2019 and this study) (**b**). For detailed case descriptions, see Table 1 and supplementary material including Supplementary Table 4. Please note the phenotypical overlap of both syndromes with respect to features of the central nervous system, eyes, and ears as well as endocrine, heart, skeletal, kidney, gastrointestinal, and genital anomalies. ^1^Kohlhase et al. (1998); Kohlhase (2007), ^2^this study (features are given in bold print), ^3^Shi et al. (2012), ^4^Webb et al. (2017), ^5^Connaughton et al. (2019), ^6^Heidet et al. (2017), ^7^Nicolaou et al. (2016), ^8^Xing et al. (2016)

Based on an index patient with caudal regression syndrome including unilateral kidney agenesis, contralateral duplex kidney with hydronephrosis, anorectal agenesis, sacral dysplasia, and malformations of the central nervous system, this study of 209 families with congenital kidney anomalies associates *DACT1* with human CAKUT and characteristic extrarenal features. Very rare non-silent *DACT1* variants were significantly more frequent in CAKUT patients of our cohort compared to controls (3.8% versus 1.7%). Moreover, CAKUT patients carrying *DACT1* variants were significantly more likely to be additionally affected by anomalies of the digestive or genital tract, skeleton (particularly the spine) or central nervous system, compared to CAKUT patients without *DACT1* variants of our cohort. Our data add *DACT1* to the list of genes underlying human syndromic CAKUT if mutated. We also establish the kidney and extrarenal phenotype spectrum caused by pathogenic *DACT1* variants, more fully defining the spectrum of features in TBS2 (Fig. 5).

DACT1 (Dapper) is required for notochord formation in *Xenopus* (Cheyette et al. 2002). *Dact1-*deficient mice have posterior malformations resembling a human caudal regression syndrome including caudal vertebrae agenesis, anorectal malformation including agenesis, and anomalies of the genitourinary system (Suriben et al. 2009; Wen et al. 2010). Kidney malformations in *Dact1-*deficient mice included fused kidneys, unilateral or bilateral kidney agenesis, cystic kidneys, and hydronephrosis, the ureters were blind-ended (Suriben et al. 2009; Wen et al. 2010). Except for bilateral kidney agenesis, all of these CAKUT phenotypes were also detected in our patients carrying *DACT1* variants, particularly frequently cystic dysplastic kidneys (comprising MCDK, cystic kidney dysplasia, and kidney hypoplasia with a single cyst). Extrarenal anomalies detected in *Dact1-*deficient mice (Suriben et al. 2009; Wen et al. 2010) and our patients with *DACT1* variants included sacral anomalies, anorectal agenesis, genital tract and bladder anomalies, i.e., a spectrum of posterior malformations. With rare exceptions, homozygous *Dact1*-deficient mice died perinatally (Suriben et al. 2009; Wen et al. 2010), thus psychomotor development, delayed in half of our patients carrying *DACT1* variants, could not be monitored. One surviving *Dact1*-deficient female adult mouse had cystic kidneys, as did more than half of our patients with *DACT1* variants, and vaginal agenesis leading to infertility (Wen et al. 2010). Infertility may particularly affect male patients with *DACT1* variants, consistent with the finding of blind-ended vas deferens in *Dact1*-deficient male mice (Wen et al. 2010), because all *DACT1* variants identified here were maternally inherited, as far as this could be determined.

The spatiotemporal expression pattern of *Dact1* detected here in murine embryos supports a direct role of DACT1 in the development of structures malformed in *Dact1*-deficient mice and patients with *DACT1* variants, such as the vertebrae, anal canal, genital tubercle, kidney and ureter, bladder, and brain. DACT1 inhibits WNT signaling, a function conserved from Xenopus to humans (Cheyette et al. 2002; Zhang et al. 2006). WNT signaling, initiated by binding of extracellular WNT ligands to the transmembrane receptor Frizzled leading to activation of Dishevelled (DVL), plays a key role in embryogenesis and kidney development (Schedl, 2007; Goggolidou 2014; Halt and Vainio 2014; Wang et al. 2018; Meng et al. 2020; https://www.wikipathways.org/index.php/Pathway:WP4150). In kidney organogenesis, WNT-mediated signals control a number of critical processes, such as intermediate mesoderm extension (WNT5A), early ureteric bud branching (WNT11), nephron induction (WNT4 and WNT9B), and the morphogenesis of the medulla (WNT7B) (Schedl 2007; Yu et al. 2009; Halt and Vainio 2014; Yun et al. 2014). In ureter development, WNTs (WNT7B, WNT9B) control smooth muscle differentiation (Trowe et al. 2012). WNT9B, expressed in the epithelial component (ureteric bud), and WNT4, expressed in the metanephric mesenchyme, are encoded by genes that can cause kidney agenesis and hypodysplasia in humans if mutated (Mandel et al. 2008; Vivante et al. 2013; Halt and Vainio 2014; Wu et al. 2017; Lemire et al. 2021; https://www.wikipathways.org/index.php/Pathway:WP5052). Here, we show that DACT1, acting downstream of these WNT ligands, is strongly expressed in the mesenchyme of the ureter and the capsular, cortical, and medullary kidney stroma from murine developmental stage E11.5 to E14.5, is involved in tubulogenesis in vitro and encodes a gene causing CAKUT in humans if mutated. Consistent with our findings that more than half of our patients with *DACT1* variants had cystic dysplastic kidneys, WNT signaling is particularly linked to cystic kidney diseases including polycystic kidney disease, nephronophthisis, medullary cystic kidney disease, and HNF1β-associated kidney anomalies (Pulkkinen et al. 2008; Goggolidou 2014). DACT1 antagonizes WNT signaling by binding DVL (Cheyette et al. 2002), and inducing DVL degradation (Zhang et al. 2006), among other mechanisms. Considering that all *DACT1* variants identified in this study encode amino acids located within the putative DVL2-binding region (Zhang et al. 2006), and showed impaired DVL2 binding, their inhibitory activity on WNT signaling may be diminished. As DVL is a central component of canonical β-catenin-dependent and non-canonical WNT signaling, both pathways may be affected by *DACT1* variants, although DACT1 appears to act mainly upstream of non-canonical planar cell polarity signaling (Suriben et al. 2009; Wen et al. 2010; Yang et al. 2013).

Recently, a heterozygous *DACT1* nonsense variant in a three-generation family was proposed to cause a TBS-like syndrome referred to as TBS2 (Webb et al. 2017). Autosomal dominant TBS1 is characterized by the triad of imperforate anus or anal stenosis in 84%, dysplastic ears in 87%, and thumb malformations in 89%, and is caused by variants in the *SALL1* (spalt like transcription factor 1) gene (Kohlhase et al. 1998; Kohlhase 2007). Functional kidney impairment with or without structural abnormalities, including polycystic kidneys, has been reported in 42% of individuals with TBS1 (Kohlhase 2007), and rare *SALL1* variants were detected in 0.5–1.4% of CAKUT patients (Hwang et al. 2014; Heidet et al. 2017; Kosfeld et al. 2018). Several anomalies in the family with TBS2 carrying a pathogenic *DACT1* variant (Webb et al. 2017) overlap with TBS1 (Kohlhase et al. 1998; Kohlhase 2007), affecting the central nervous system, the ears, the endocrine system, the kidneys, the gastrointestinal and genital tract, and the skeleton, whereas thumb abnormalities were not observed (Fig. 5). Here, we report eight new families with heterozygous *DACT1* variants, emphasizing the importance of kidney anomalies and supporting the observation of a characteristic phenotype spectrum additionally involving the skeleton (particularly the spine), the digestive and genital tract, and the central nervous system in TBS2, similar to that in TBS1 (Fig. 5). This combination of phenotypic features in TBS2 is confirmed when combining our data with that from the literature totaling 26 patients from 19 families with very rare *DACT1* variants, investigated because of neural tube defects (Shi et al. 2012), Müllerian duct (Xing et al. 2016) or kidney anomalies (Nicolaou et al. 2016; Heidet et al. 2017; Connaughton et al. 2019; this study) or TBS-like features (Webb et al. 2017). Families carrying *DACT1* variants recurrently presented with anomalies of the kidney (12/19, 63%), skeleton (12/19, 63%), central nervous system (10/19, 53%), genital tract (4/19, 21%), lung (3/19, 16%), and distal digestive tract (2/19, 10.5%) (Fig. 5; Supplementary Table 4). Of note, congenital kidney anomalies were observed in almost two-thirds of families. Thus, similar to the mouse model, in which *Dact1* deficiency is fully penetrant for kidney anomalies (Suriben et al. 2009; Wen et al. 2010), CAKUT seem to be a major feature in patients with rare heterozygous *DACT1* variants.

However, incomplete penetrance with respect to CAKUT and extrarenal phenotypes and variable expressivity is observed in individuals carrying heterozygous *DACT1* variants that may be unaffected or show variable features of TBS2, leading to miscarriage in the worst case (Fig. 1, Supplementary Table 4). Similarly, few *Dact1*-deficient mice survive postnatally and present with milder malformations, while most die perinatally due to severe developmental defects (Suriben et al. 2009; Wen et al. 2010). Incomplete penetrance is commonly observed in autosomal dominant familial CAKUT, and environmental factors or epigenetic alterations may contribute to CAKUT pathogenesis and severity of defects (Sanna-Cherchi et al. 2018; van der Ven et al. 2018b; Nigam et al. 2019). Similar to findings in patients with heterozygous *DACT1* variants, heterozygous variants in *SALL1* have been identified in patients with typical features of TBS1, but also in patients with isolated CAKUT (Hwang et al. 2014; Heidet et al. 2017; Kosfeld et al. 2018).

In conclusion, we identified very rare heterozygous missense variants in the DVL2 interaction region of *DACT1* in 3.8% of CAKUT families. We provide further evidence that deleterious *DACT1* variants and *Dact1* deficiency cause kidney anomalies and a defined spectrum of extrarenal malformations in mice and humans, referred to as TBS2. When identified in CAKUT patients with features of TBS2, rare *DACT1* variants may be considered causative, especially in cases without *SALL1* variants. Kidney ultrasound is warranted in patients carrying rare *DACT1* variants since two-thirds of families described so far present with kidney agenesis, duplex/fused or (multi)cystic (hypo)dysplastic kidneys with hydronephrosis.

## Supplementary Information

Below is the link to the electronic supplementary material.

Supplementary file1 (PDF 1704 KB)

Supplementary file2 (XLSX 50 KB)
