# Supplementary material for: Heterozygous variants in the DVL2 interaction region of DACT1 cause CAKUT and features of Townes–Brocks syndrome 2
Source: Hum Genet. 2022 Sep 6;142(1):73–88. doi: 10.1007/s00439-022-02481-6 (PMC9839807; doi:10.1007/s00439-022-02481-6)
Supplement: Supplementary file 1 — Supplementary file1 (PDF 1704 KB) [file 439_2022_2481_MOESM1_ESM.pdf]

Supplementary material

Heterozygous variants in the DVL2 interaction region of *DACT1* cause CAKUT and features of Townes-Brocks syndrome 2

Anne Christians[1], Esra Kesdiren[1], Imke Hennies[2], Alejandro Hofmann[3], Mark-Oliver Trowe[4], Frank Brand[1], Helge Martens[1], Ann Christin Gjerstad[5], Zoran Gucev[6], Matthias Zirngibl[7], Robert Geffers[8], Tomáš Seeman[9], Heiko Billing[7], Anna Bjerre[5], Velibor Tasic[6], Andreas Kispert[4], Benno Ure[3], Dieter Haffner[2], Jens Dingemann[3], Ruthild G. Weber[1]

[1]Department of Human Genetics, Hannover Medical School, Hannover, Germany;

[2]Department of Pediatric Kidney, Liver and Metabolic Diseases, Hannover Medical School, Hannover, Germany;

[3]Department of Pediatric Surgery, Hannover Medical School, Hannover, Germany;

[4]Institute of Molecular Biology, Hannover Medical School, Hannover, Germany;

[5]Division of Paediatric and Adolescent Medicine, Oslo University Hospital, Oslo, Norway;

[6]Pediatric Nephrology, University Children's Hospital, Skopje, North Macedonia;

[7]Pediatric Nephrology, University Children's Hospital, Tübingen, Germany;

[8]Genome Analytics Research Group, Helmholtz Centre for Infection Research, Braunschweig, Germany;

[9]Department of Pediatrics, 2nd Faculty of Medicine, Charles University Prague, Czech Republic

Correspondence to: Ruthild G. Weber, Department of Human Genetics OE 6300, Hannover Medical School, Carl-Neuberg-Str. 1, 30625 Hannover, Germany, Phone: +49 511 5327751, Fax: +49 511 53218520, E-mail: weber.ruthild@mh-hannover.de

Case reports of patients carrying *DACT1* variants

Very rare heterozygous *DACT1* missense variants were identified in index patients V005-II.04, N062-II.03, C003-II.01, T004-II.03, H452-II.01, H402-II.01, B036-II.01, N032-II.01, and members of their families.

V005-II.04 (born in 2016): NM_016651.5(*DACT1*):c.1100C>A p.(Thr367Lys)

The boy is the fourth child of non-consanguineous Kurdish parents (Fig. 2a-h). Due to a prenatally diagnosed hydrocephalus, birth was induced at gestational age of 38 2/7 weeks. At birth, weight was 2580 g (4th percentile), body length was 48 cm (15th percentile) and head circumference was 34.8 cm (50th percentile). After birth, the boy presented with additional internal and external malformations. Kidney ultrasound was notable for left-sided kidney agenesis. Diagnostic work-up showed a right-sided malrotated duplex kidney with hydronephrosis and primary obstructive megaureter without urodynamic relevant stenosis. Vesicoureteral reflux was not observed. Estimated glomerular filtration rate (eGFR) was normal. Additionally, the patient presented with a caudal regression syndrome with missing coccyx, sacral dysplasia, anorectal agenesis, and rectourethral fistula. A posterior sagittal anorectoplasty was performed at the age of 4 months. Internal and external genitalia were unremarkable. The hydrocephalus internus (ventricles I-III), confirmed after birth, was found to result from an aqueductal stenosis. Additionally, an agenesis of the septum pellucidum and the cerebellar vermis were diagnosed. The patient also presented with a syringohydromyelia at T11-T12 and an intraspinal dermoid cyst at L2-L3. Newborn screening and examination of the eyes, heart, liver, gallbladder, and spleen were unremarkable. Kidney ultrasound of the parents was also unremarkable.

N062-II.03 (born in 2004): NM_016651.5(*DACT1*):c.1100C>A p.(Thr367Lys)

The girl is the third daughter of a Turkish couple that are second cousins. The fetus was prenatally diagnosed with a right-sided multicystic dysplastic kidney, which had regressed completely during the first two years of life. The girl had a normal, slightly enlarged left kidney.

Estimated GFR and blood pressure were normal. There was no history of kidney malformation in the family. Kidney ultrasound of the parents and siblings was not performed.

C003-II.01 (born in 2000): NM_016651.5(*DACT1*):c.1703G>A p.(Arg568Lys)

The boy is a child of Czech parents. He presented with bilateral cystic kidney dysplasia as well as intrauterine growth retardation. Additionally, he was diagnosed with epilepsy, mental retardation and autism. No further information was available.

T004-II.03 (born in 2008): NM_016651.5(*DACT1*):c.1771C>T p.(Leu591Phe)

The boy is a child of non-consanguineous parents from Greece (Fig. 2i, j). He was born at a gestational age of 38 weeks (birth weight: 2.91 kg, 20th percentile) with no obvious malformations noted at birth. When he initially presented at the age of 4 ½ years, the boy had a short stature with a body length of <3rd percentile, body weight at 20th percentile, head circumference at 8th percentile. Ultrasound examinations showed bilateral severely dysplastic kidneys with extensive hydronephrosis (Fig. 2i). Videourodynamics revealed a massively distended urinary bladder (megacystis) with bilateral high-grade vesicoureteral reflux (Fig. 2j). There was no evidence for posterior urethral valves. At the age of six years, an appendicovesicostomy (Mitrofanoff procedure) was performed and clean intermittent catheterization initiated. At 4 ½ years, eGFR was 50 ml/min/1.73m² (i.e. chronic kidney disease stage 3). Over 5 years, a continuous decline in eGFR was observed resulting in end-stage kidney disease. At 9 ½ years, kidney replacement therapy (hemodialysis) was initiated. The patient is attending a school for children with special needs. An intelligence test at the age of 10 years revealed a learning disability (overall IQ of 71) with low speech comprehension (IQ of 65), reduced information processing speed (IQ of 81), and impaired auditory working memory (IQ of 82) and ability to reason (IQ of 79). He has a healthy older sister, a healthy twin brother and a younger brother (T004-II.04) who also presented with megacystis. The parents were clinically unremarkable.

H452-II.01 (born in 2006): NM_016651.5(*DACT1*):c.1890G>T p.(Lys630Asn)

The girl is the first child of North Macedonian parents (Fig. 2k). Kidney ultrasound revealed right-sided kidney hypoplasia without focal defects (Fig. 2k), and with partial function (40%) on dimercaptosuccinic acid (DMSA) kidney scan. Additionally, she presented with high arched palate. Kidney and bladder ultrasound of both parents was unremarkable.

H402-II.01 (born in 2002): NM_016651.5(*DACT1*):c.1931G>A p.(Gly644Asp)

The girl is the first child of a North Macedonian couple (Fig. 2l). Kidney ultrasound revealed right-sided kidney hypoplasia with a cyst in the upper pole (Fig. 2l), which was confirmed by magnetic resonance imaging urography. By voiding cystourethrogram, vesicoureteral reflux was diagnosed in the right distal ureter. The left kidney was normal. Additionally, she presented with dextroposition of the uterus and a cystic/tumorous formation at the right ovarium on magnetic resonance imaging scan. Ultrasonography of the kidney and bladder of her father and siblings was normal. Her mother had a putative left-sided non-obstructive duplex kidney.

B036-II.01 (born in 2015): NM_016651.5(*DACT1*):c.2005C>G p.(Pro669Ala)

The boy is the only child of a German woman inseminated with donated sperm (Fig. 2m, n). The boy was prenatally diagnosed with left-sided multicystic dysplastic kidney. Further evaluation after birth showed a single right kidney with normal function and a left-sided dilated residual ureter ending in a ureterocele in the bladder (Fig. 2m, n). The patient has no other malformations or stigmata. Kidney ultrasound of his mother was normal. There is no history of kidney malformations in the maternal side of the family.

N032-II.01 (born in 2014): NM_016651.5(*DACT1*):c.2468T>G p.(Leu823Arg)

The girl is the first child of a non-consanguineous Afro-American couple (Fig. 2o-q). Her birth weight was 3125 g. The post-natal period was unremarkable. Evaluation by DMSA kidney scan and kidney ultrasound showed a normal sized single right kidney and residues of a left-sided multicystic dysplastic kidney (Fig. 2p, q). Estimated GFR and blood pressure were normal. X-

ray examination of the spine revealed a lumbarization of S1 (Fig. 2o). She was also diagnosed with high arched palate, narrow forehead, epicanthus, bone protrusion between the eyes, and flattened, almost concave temporal bones. Additionally, she presented with delayed psychomotor development and atypical autism. The mother was also diagnosed with high arched palate, while kidney ultrasound was unremarkable. There is no history of kidney malformation in the family.

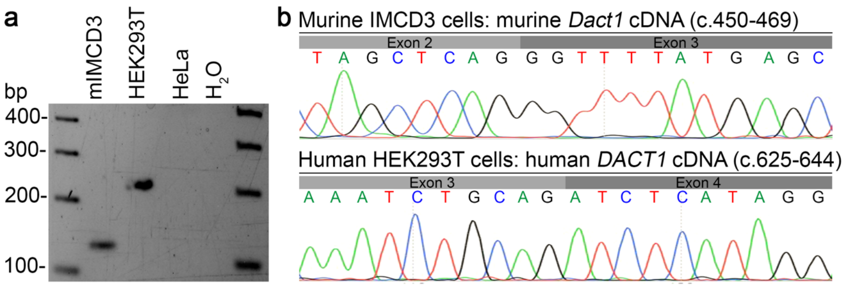

Supplementary Fig. 1 Qualitative *Dact1* mRNA analysis confirming the presence of murine *Dact1*-specific sequences in RNA samples from murine inner medullary collecting duct (mIMCD3) cells, and human *DACT1*-specific sequences in RNA samples from human embryonic kidney 293T (HEK293T) cells (positive control according to www.proteinatlas.org), and the absence in RNA samples from human HeLa cells (negative control according to www.proteinatlas.org). After first-strand cDNA synthesis from total RNA, specific oligonucleotides (Supplementary Table 3) were used to amplify an exon-spanning fragment of the murine *Dact1* cDNA sequence (NM_021532.4, c.390-518) or the human *DACT1* cDNA sequence (NM_016651.6, c.487-709), respectively. **a** By gel electrophoresis, a PCR product of 129 bp consistent with the expected murine *Dact1* cDNA band was detected in mIMCD3 cells, and a PCR product of 223 bp consistent with the expected human *DACT1* cDNA band was observed in HEK293T cells. As expected, no PCR product was detected in HeLa cells. **b** Each PCR product shown in (**a**) was subjected to direct sequencing. The obtained electropherograms confirmed the presence of sequences specific for murine *Dact1* cDNA in mIMCD3 cells and human *DACT1* cDNA in HEK293T cells.

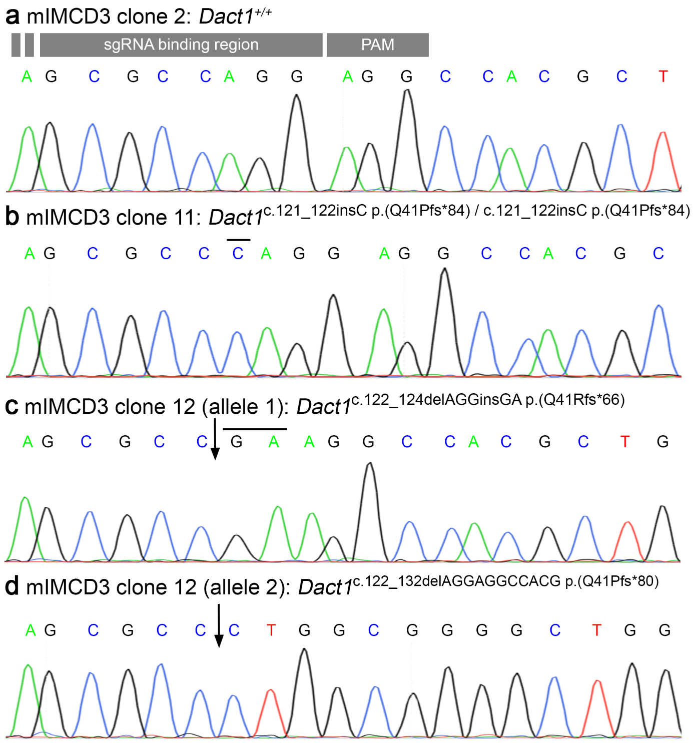

Supplementary Fig. 2 Electropherograms of the single guide RNA (sgRNA) on-target site in *Dact1* exon 1 of murine inner medullary collecting duct (mIMCD3) cells modified by CRISPR/Cas9 technique, as previously described (Ran et al. 2013), to generate *Dact1* knockout cells. **a-d** We selected a clone with no mutational event in *Dact1* (clone 2, *Dact1*$^{+/+}$, **a**) as a control. A clone with a homozygous frameshift variant (clone 11, *Dact1*$^{-/-}$, **b**) and a clone with different frameshift variants on both alleles (clone 12, *Dact1*$^{-/-}$, separated alleles are shown in **c** and **d**), all of which were predicted to result in a premature stop codon and a non-functional protein, were selected as *Dact1* knockout cells. The position of deletions is indicated by black arrows, inserted nucleotides are marked by black lines. In these three clones, 13 exonic off-target sites were sequenced to ascertain absence of mutation (Supplementary Table 3). The protospacer adjacent motif (PAM) for Cas9 binding and parts of the sgRNA target region are shown in (**a**).

Supplementary Table 1 Spectrum of kidney anomalies with or without urinary tract anomalies in 209 analyzed patients

Kidney anomalies with or without anomalies of the urinary tract	Frequency
Unilateral kidney agenesis	25/209
- with contralateral cystic kidney dysplasia	2/25
- with contralateral kidney dysplasia	9/25
- with contralateral kidney dysplasia and obstructive megaureter	1/25
- with contralateral duplex kidney	3/25
- with contralateral crossed ectopic kidney	1/25
Unilateral MCDK	19/209
- with contralateral cystic kidney dysplasia	1/19
- with contralateral duplex kidney	1/19
- with contralateral ureterocele	1/19
- with contralateral PUJO	1/19
Bilateral cystic kidney dysplasia	28/209
- with bilateral primary obstructive megaureter	1/28
- with megacystis	1/28
- with PUV	2/28
Unilateral cystic kidney dysplasia	3/209
Bilateral kidney dysplasia	78/209
- with bilateral kidney hypoplasia	1/78
- with unilateral kidney hypoplasia	1/78
- with bilateral primary obstructive megaureter	1/78
- with bilateral PUJO	1/78
- with bilateral PUJO and primary obstructive megaureter	1/78
- with unilateral PUJO	1/78
- with megacystis	4/78
- with megacystis and PUV	1/78
- with PUV	18/78
Unilateral kidney dysplasia	31/209
- with contralateral duplex kidney and hydroureter	1/31
- with kidney ectopia	1/31
- with bilateral PUJO	1/31
- with unilateral PUJO	1/31
- with unilateral ureterocele	1/31
- with PUV	1/31
Bilateral kidney hypoplasia	2/209
- with PUV	1/2
Unilateral kidney hypoplasia	11/209
- with kidney ectopia	2/11
- with bilateral primary obstructive megaureter	1/11
Bilateral duplex kidney	2/209
- with bilateral ureteroceles	1/2
Unilateral duplex kidney	3/209
- with contralateral triple kidney and bilateral kidney dysplasia	1/3
- with bilateral PUJO	1/3
Horseshoe kidney	5/209
- with primary obstructive megaureter	1/5
Crossed fused kidney	1/209
Kidney ectopia	1/209

MCDK, multicystic dysplastic kidney; PUJO, pelviureteric junction obstruction; PUV, posterior urethral valves

Supplementary Table 2 Analysis of WES data from patient V005-II.04 using a candidate gene-based strategy identified a very rare heterozygous *DACT1* missense variant

Filtering steps[a]	Number of variants
Total variants in exome obtained from peripheral blood with the following quality scores: read depth ≥50, call quality ≥50, allele fraction ≥45%	14,128
Non-silent variants, i.e. splice site (up to 20 bases into intron), frameshift, in-frame indels, stop gained/lost, and non-synonymous missense variants, are retained	4,173
Population filtering: very rare variants (minor allele frequency ≤0.0005 in the 1000 Genomes Project, ExAC database, gnomAD database, or NHLBI ESP exomes) are retained[b]	158
Comparison with identically generated exome data of unrelated in-house control individuals (n=137), variants not present in controls are retained	74
Variants in genes reported as human isolated or syndromic CAKUT (candidate) genes or murine CAKUT (candidate) genes according to our in-house gene list (n=455 genes)[c] are retained	1 (*DACT1*)

[a]Prioritization was performed using Ingenuity Variant Analysis (Qiagen, Hilden, Germany)

[b]According to 1000 Genomes Project data (https://www.internationalgenome.org/), Exome Aggregation Consortium (ExAC; https://gnomad.broadinstitute.org/), Genome Aggregation Database (gnomAD; https://gnomad.broadinstitute.org/), NHLBI Exome Sequencing Project (NHLBI ESP; https://evs.gs.washington.edu/EVS/)

[c]According to Blackburn et al. 2019; Connaughton et al. 2019; Heidet et al. 2017; Jain and Chen 2019; Kim et al. 2019; Kitzler et al. 2019; Kosfeld et al. 2018; Nicolaou et al. 2016; van der Ven et al. 2018a; van der Ven et al. 2018b; Vivante and Hildebrandt 2016

Supplementary Table 3 Oligonucleotides used for different applications, as indicated

**Designation**	**Sequence (5' to 3') and possible 5' phosphorylation (5'-PHOS)**
Amplification and Sanger sequencing of human *DACT1* coding exons	
hDACT1_Ex1_F	GCA GGA CTC GAG GGC TTC TA
hDACT1_Ex1_R	ATC TCC CCA GCC AGA GTC AA
hDACT1_Ex2_F	TAG TGA ACT CTT CAG ATG GG
hDACT1_Ex2_R	GGA AAC AAA CTC CAT TGC C
hDACT1_Ex3_F	TTC ATT TTC CTG TTA AGC G
hDACT1_Ex3_R	AGT GAA AGG TGC TGG AGC
hDACT1_Ex4a_F	ATT GGC CAA AGA GTC AAG C
hDACT1_Ex4a_R	GCA GGT GCT TAC TCT GAA GG
hDACT1_Ex4b_F	ATA CCT TCT CTG AAC AAT GGG
hDACT1_Ex4b_R	CCG CCG GTA GTC AGT TCG
hDACT1_Ex4c_F	GCA AGA AGT GTC GCT TCC C
hDACT1_Ex4c_R	CAC CAC CTT TTT AAT TTT GG
Generation of a *pcDNA3.1-Myc-DACT1* expression construct	
InFusion-hDACT1_F	TGG AAT TCT GCA GAT ATC CAA AGC CGA GTC CGG CCG
InFusion-hDACT1_R	GCC AGT GTG ATG GAT ATC TCA AAC CGT CGT CAT CAG TTT C
Site-directed mutagenesis of the *pcDNA3-Myc-DACT1* expression construct	
hDACT1_c1100C>A_F	GAC CCC AAG AAA GGG CTT CTG AG
hDACT1_c1100C>A_R_P	AGC GTT CAC GCT GGT TCT TGG (5'-PHOS)
hDACT1_c1703G>A_F	CCA GTG TCA AGC TCC ACC GGG
hDACT1_c1703G>A_R_P	GCT GCT GCC CCG GGA TAA ACT G (5'-PHOS)
hDACT1_c1771C>T_F	CCA GCC TTC CAG GGG CTG GAG
hDACT1_c1771C>T_R_P	GCC GCG GTG CTT CAG GCT G (5'-PHOS)
hDACT1_c1890G>T_F_P	TCC AAT GGG AGG AAG AGT GGG (5'-PHOS)
hDACT1_c1890G>T_R	GGA GGC TTT CTT GAG TTT CTT ATT TG
hDACT1_c1931G>A_F	GTT CCC GAC AGG CCC GCG GGC G
hDACT1_c1931G>A_R_P	ACC AGC CTC GGG CCC GCC CCC (5'-PHOS)
hDACT1_c2005C>G_F	GTG GCC AAA GCT AAG CAC AAG CGA ACT G
hDACT1_c2005C>G_R_P	CAC CGC CTC CCG TCC GTG GC (5'-PHOS)
hDACT1_c2468T>G_F	GGT CTG GCT CTT TGA AAC TGA TGA CG
hDACT1_c2468T>G_R_P	GAA AGC GGC GGA TCT TCT TCT TGA GG (5'-PHOS)
Qualitative mRNA expression analysis of murine *Dact1* and human *DACT1*	
mDact1_Ex2_Fwd	TGA CCT GAG ACT GGA TGT GG
mDact1_Ex3_Rev	AAC ACG GAG TTG GAG GAG TT
hDACT1_Ex3_Fwd	GAG CTG AGT GAT GGG GCT TC
hDACT1_Ex4_Rev	AGA GGG AAC GGC AAA CTG C
Generation of a *pSpCas9(BB)-2A-GFP-Dact1-sgRNA* construct	
mDact1_sgRNA_Ex1_F	CAC CGC GTA CCC GCG AGC GCC AGG
mDact1_sgRNA_Ex1_R	AAA CCC TGG CGC TCG CGG GTA CGC
Amplification, cloning, sequencing of murine *Dact1* exon 1 including the CRISPR/Cas9 target site	
mDact1_Ex1_BamHI_F	ATA TGG ATC CAT GAA GCC GGA CGC AG
mDact1_Ex1_EcoRI_R	TAT AGA ATT CCC ACA GCC AGA GGC C
Amplification and sequencing of predicted Cas9 off-target sites on DNA of selected murine IMCD3 cell clones	
mDact1-sgRNA_Rgma_F	TCT CGG AAC TGC AAG CTA CG
mDact1-sgRNA_Rgma_R	ACT CAG TAG CGA CCC GGG
mDact1-sgRNA_Btbd3_F	AAG ATT CCC TCT AAA GCG CC

mDact1-sgRNA_Btbd3_R	GAA CTC AGG GAT CCA GAG CC
mDact1-sgRNA_a_F	CTA CTC CTG GCC ACC CTA GT
mDact1-sgRNA_a_R	GCC ACG ATA GAA ACA GAG G
mDact1-sgRNA_Amdhd1_F	GTA CTG AGT GAG CTC CGC AG
mDact1-sgRNA_Amdhd1_R	GGC CAC GTA CAT GGA CAT TC
mDact1-sgRNA_Nudt19_F	GAT GTC AGG CGT GCA GTC TA
mDact1-sgRNA_Nudt19_R	CTT CTC AGG AGC CCA GTC AG
mDact1-sgRNA_Ddah1_F	CCA TGT GAG GCG TGC AC
mDact1-sgRNA_Ddah1_R	GGG TTT GAG TCG GTG GC
mDact1-sgRNA_Slc4a9_F	CAC TGG GTC AAT GGC AGC
mDact1-sgRNA_Slc4a9_R	TGG GAC AGG CTC TGG AAG
mDact1-sgRNA_Zcchc3_F	GAC AGG CAC TTT CGT CTC C
mDact1-sgRNA_Zcchc3_R	GAC GAA ACT GGC AGC GGT
mDact1-sgRNA_Pbld2_F	TTC ACT GCG ACA GCT TTC
mDact1-sgRNA_Pbld2_R	GAA CAC CAG AGT TGC AGG
mDact1-sgRNA_Cpxm2_F	GTC TTC ATG CAT TGA AGG C
mDact1-sgRNA_Cpxm2_R	CTG TCC CAC CCA CCA TC
mDact1-sgRNA_Prdm11_F	TTC ATA TAC TGG TCC ACC ACC
mDact1-sgRNA_Prdm11_R	GTA CAC GGT TCA GTC CTC AC
mDact1-sgRNA_Pbld1_F	CGT TCA CTG CGA CAG CTT TC
mDact1-sgRNA_Pbld1_R	CTC CTT TGG AAC ACC AGA GC
mDact1-sgRNA_Gid4_F	GTG AGC GCG TGT GTA TG
mDact1-sgRNA_Gid4_R	CCT CTA CGT CGT ACG AGT TC

Ex, exon; F or Fwd, forward; P, primer; R or Rev, reverse; sgRNA, single guide RNA

Supplementary Table 4 Very rare (MAF ≤0.0005) heterozygous *DACT1* variants and patient phenotypes reported here and previously

Nucleotide alteration[a]	Deduced protein change[a]	MAF[b]	Case, gender	Anomalies of the kidneys and urinary tract	Extrarenal anomalies	References
c.49C>G	p.(Pro17Ala)	-	P18-56, female	Kidney hypodysplasia (bilat)	Scoliosis	Connaughton et al. 2019
c.133C>T	p.(Arg45Trp)	0.000245	Fetus, female	-	Anencephalus, spina bifida aperta, exophthalmos (l), lung lobe deformity	Shi et al. 2012
c.169G>A	p.(Glu57Lys)	-	K65, fetus, male	Multicystic kidney dysplasia (bilat)	ND	Heidet et al. 2017
c.425A>G	p.(Asp142Gly)	-	Fetus, female	-	Complete anencephalus, spina bifida aperta, absence of neck, thorax deformity, overlength of upper limbs, lung lobe deformity, atelectasis	Shi et al. 2012
c.1068C>A	p.(Asn356Lys)	-	Fetus, female	-	Anencephalus, spina bifida aperta, atrial septal defect, lung lobe deformity (l), adrenal dysplasia (bilat), atelectasis	Shi et al. 2012
c.1084G>A	p.(Val362Met)	0.000009	Female	-	Didelphic uterus	Xing et al. 2016
c.1100C>A	p.(Thr367Lys)	0.000333	V005-II.04, male	Kidney agenesis (l); malrotated duplex kidney, hydronephrosis, primary obstructive megaureter (r), neurogenic bladder	Caudal regression syndrome with missing coccyx, sacral dysplasia, syringohydromyelia, intraspinal dermoid cyst, anorectal agenesis with rectourethral fistula, agenesis of the septum pellucidum, triventricular hydrocephalus internus due to an aqueductal stenosis, agenesis of the cerebellar vermis	This study
			N062-II.03, female	MCDK (r)	-	
c.1169T>C	p.(Val390Ala)	0.000224	Female	ND	Mullerian duct anomalies	Xing et al. 2016
c.1256G>A	p.(Trp419*)	-	III.4, female	Crossed fused renal ectopia, VUR	Imperforate anus, rectovaginal fistula, auricular cupping and overfolding of the superior helix (bilat)	Webb et al. 2017
			III.2, male	Crossed fused renal ectopia, VUR	Imperforate anus, hypospadias, attention deficit hyperactivity disorder, ear cupping (r), overfolding of the superior helix (bilat)	

			II.2, female	- (kidney US)	Spina bifida occulta, scoliosis, Graves' disease, ear cupping (bilat)	
			II.3, female	- (kidney US)	Severe microtia (l)	
			I.4, female	- (kidney US)	Severe microtia (l), bifid uterus, Hashimoto's thyroiditis	
c.1703G>A	p.(Arg568Lys)	-	C003-II.01, male	Cystic kidney dysplasia (bilat)	Intrauterine growth retardation, mental retardation, autism, epilepsy	This study
c.1771C>T	p.(Leu591Phe)	0.000048	T004-II.03, male	Kidney dysplasia, hydronephrosis, megaureter, VUR (all bilat), megacystis	Short stature, learning disability (overall IQ of 71)	This study
			T004-II.04, male	Megacystis, normal kidneys (kidney US)	-	
c.1890G>T	p.(Lys630Asn)	0.000354	H452-II.01, female	Kidney hypoplasia (r)	High arched palate	This study
c.1931G>A	p.(Gly644Asp)	0.000085	H402-II.01, female	Kidney hypoplasia with a cyst in the upper pole, VUR (all r)	Dextroposition of the uterus, ovarian cyst (r)	This study
			H402-I.02, female	Non-obstructive duplex kidney (l) (putative)	-	
c.2005C>G	p.(Pro669Ala)	-	B036-II.01, male	MCDK, dilated residual ureter ending in ureterocele (all l)	-	This study
c.2105T>G	p.(Val702Gly)	-	Fetus, female	-	Hypoxic ischemic encephalopathy, lumbosacral closed spina bifida	Shi et al. 2012
c.2423C>A	p.(Thr808Asn)	0.000009	Fetus, female	-	Occipital meningoencephalocele, thorax deformity	Shi et al. 2012
c.2468T>G	p.(Leu823Arg)	-	N032-II.01, female	MCDK (l)	Lumbarization of S1, high arched palate, narrow forehead, bone protrusion between the eyes, flattened/concave temporal bones, epicanthus,	This study

					delayed psychomotor development, autism (atypical)	
			N032-I.02, female	- (kidney US)	High arched palate	
c.2483G>A	p.(Gly828Asp)	-	Female	Duplex collecting system (unilateral), VUR, ureterocele	ND	Nicolaou et al. 2016

[a]Reference sequence: NM_016651.5; genome build: GRCh37/hg19

[b]According to gnomAD controls v2.1.1, total population (https://gnomad.broadinstitute.org/)

[c]ClinVar: https://www.ncbi.nlm.nih.gov/clinvar/

Abbreviations: bilat, bilateral; l, left; MAF, minor allele frequency; MCDK, multicystic dysplastic kidney; ND, not determined; r, right; kidney US, kidney ultrasound; VUR, vesicoureteral reflux; -, not present

Supplementary Table 5 Details of 355 very rare (MAF ≤0.0005) non-silent variants in *DACT1* affecting amino acids located outside or inside of the DVL2 interaction region carried by gnomAD controls v2.1.1, total population. See separate .xlsx-file.

References

Blackburn ATM, Bekheirnia N, Uma VC, Corkins ME, Xu Y, Rosenfeld JA, Bainbridge MN, Yang Y, Liu P, Madan-Khetarpal S, Delgado MR, Hudgins L, Krantz I, Rodriguez-Buritica D, Wheeler PG, Gazali LA, Mohamed Saeed Mohamed Al Shamsi A, Gomez-Ospina N, Chao HT, Mirzaa GM, Scheuerle AE, Kukolich MK, Scaglia F, Eng C, Willsey HR, Braun MC, Lamb DJ, Miller RK, Bekheirnia MR (2019) DYRK1A-related intellectual disability: a syndrome associated with congenital anomalies of the kidney and urinary tract. Genet Med 21:2755–2764. https://doi.org/10.1038/s41436-019-0576-0

Connaughton DM, Kennedy C, Shril S, Mann N, Murray SL, Williams PA, Conlon E, Nakayama M, van der Ven AT, Ityel H, Kause F, Kolvenbach CM, Dai R, Vivante A, Braun DA, Schneider R, Kitzler TM, Moloney B, Moran CP, Smyth JS, Kennedy A, Benson K, Stapleton C, Denton M, Magee C, O'Seaghdha CM, Plant WD, Griffin MD, Awan A, Sweeney C, Mane SM, Lifton RP, Griffin B, Leavey S, Casserly L, de Freitas DG, Holian J, Dorman A, Doyle B, Lavin PJ, Little MA, Conlon PJ, Hildebrandt F (2019) Monogenic causes of chronic kidney disease in adults. Kidney Int 95:914–928. https://doi.org/10.1016/j.kint.2018.10.031

Heidet L, Moriniere V, Henry C, De Tomasi L, Reilly ML, Humbert C, Alibeu O, Fourrage C, Bole-Feysot C, Nitschke P, Tores F, Bras M, Jeanpierre M, Pietrement C, Gaillard D, Gonzales M, Novo R, Schaefer E, Roume J, Martinovic J, Malan V, Salomon R, Saunier S, Antignac C, Jeanpierre C (2017) Targeted Exome Sequencing Identifies PBX1 as Involved in Monogenic Congenital Anomalies of the Kidney and Urinary Tract. J Am Soc Nephrol 28:2901–2914. https://doi.org/10.1681/ASN.2017010043

Jain S, Chen F (2019) Developmental pathology of congenital kidney and urinary tract anomalies. Clin Kidney J 12:382–399. https://doi.org/10.1093/ckj/sfy112

Kim JH, Park EY, Chitayat D, Stachura DL, Schaper J, Lindstrom K, Jewett T, Wieczorek D, Draaisma JM, Sinnema M, Hoeberigs C, Hempel M, Bachman KK, Seeley AH, Stone JK, Kong HK, Vukadin L, Richard A, Shinde DN, McWalter K, Si YC, Douglas G, Lim ST, Vissers L, Lemaire M, Ahn EE (2019) SON haploinsufficiency causes impaired pre-

mRNA splicing of CAKUT genes and heterogeneous renal phenotypes. Kidney Int 95:1494–1504. https://doi.org/10.1016/j.kint.2019.01.025

Kitzler TM, Schneider R, Kohl S, Kolvenbach CM, Connaughton DM, Dai R, Mann N, Nakayama M, Majmundar AJ, Wu CW, Kari JA, El Desoky SM, Senguttuvan P, Bogdanovic R, Stajic N, Valivullah Z, Lek M, Mane S, Lifton RP, Tasic V, Shril S, Hildebrandt F (2019) COL4A1 mutations as a potential novel cause of autosomal dominant CAKUT in humans. Hum Genet 138:1105–1115. https://doi.org/10.1007/s00439-019-02042-4

Kosfeld A, Martens H, Hennies I, Haffner D, Weber RG (2018) Kongenitale Anomalien der Nieren und ableitenden Harnwege (CAKUT). medizinische genetik 30:448–460. https://doi.org/10.1007/s11825-018-0226-y

Nicolaou N, Pulit SL, Nijman IJ, Monroe GR, Feitz WF, Schreuder MF, van Eerde AM, de Jong TP, Giltay JC, van der Zwaag B, Havenith MR, Zwakenberg S, van der Zanden LF, Poelmans G, Cornelissen EA, Lilien MR, Franke B, Roeleveld N, van Rooij IA, Cuppen E, Bongers EM, Giles RH, Knoers NV, Renkema KY (2016) Prioritization and burden analysis of rare variants in 208 candidate genes suggest they do not play a major role in CAKUT. Kidney Int 89:476–486. https://doi.org/10.1038/ki.2015.319

Ran FA, Hsu PD, Wright J, Agarwala V, Scott DA, Zhang F (2013) Genome engineering using the CRISPR-Cas9 system. Nature protocols 8:2281–2308. https://doi.org/10.1038/nprot.2013.143

Shi Y, Ding Y, Lei YP, Yang XY, Xie GM, Wen J, Cai CQ, Li H, Chen Y, Zhang T, Wu BL, Jin L, Chen YG, Wang HY (2012) Identification of novel rare mutations of DACT1 in human neural tube defects. Hum Mutat 33:1450–1455. https://doi.org/10.1002/humu.22121

van der Ven AT, Connaughton DM, Ityel H, Mann N, Nakayama M, Chen J, Vivante A, Hwang DY, Schulz J, Braun DA, Schmidt JM, Schapiro D, Schneider R, Warejko JK, Daga A, Majmundar AJ, Tan W, Jobst-Schwan T, Hermle T, Widmeier E, Ashraf S, Amar A, Hoogstraaten CA, Hugo H, Kitzler TM, Kause F, Kolvenbach CM, Dai R, Spaneas L, Amann K, Stein DR, Baum MA, Somers MJG, Rodig NM, Ferguson MA, Traum AZ,

Daouk GH, Bogdanovic R, Stajic N, Soliman NA, Kari JA, El Desoky S, Fathy HM, Milosevic D, Al-Saffar M, Awad HS, Eid LA, Selvin A, Senguttuvan P, Sanna-Cherchi S, Rehm HL, MacArthur DG, Lek M, Laricchia KM, Wilson MW, Mane SM, Lifton RP, Lee RS, Bauer SB, Lu W, Reutter HM, Tasic V, Shril S, Hildebrandt F (2018a) Whole-Exome Sequencing Identifies Causative Mutations in Families with Congenital Anomalies of the Kidney and Urinary Tract. J Am Soc Nephrol 29:2348–2361. https://doi.org/10.1681/ASN.2017121265

van der Ven AT, Vivante A, Hildebrandt F (2018b) Novel Insights into the Pathogenesis of Monogenic Congenital Anomalies of the Kidney and Urinary Tract. J Am Soc Nephrol 29:36–50. https://doi.org/10.1681/ASN.2017050561

Vivante A, Hildebrandt F (2016) Exploring the genetic basis of early-onset chronic kidney disease. Nat Rev Nephrol 12:133–146. https://doi.org/10.1038/nrneph.2015.205

Webb BD, Metikala S, Wheeler PG, Sherpa MD, Houten SM, Horb ME, Schadt EE (2017) Heterozygous Pathogenic Variant in DACT1 Causes an Autosomal-Dominant Syndrome with Features Overlapping Townes-Brocks Syndrome. Hum Mutat 38:373–377. https://doi.org/10.1002/humu.23171

Xing Q, Xu Z, Zhu Y, Wang X, Wang J, Chen D, Xu Y, He X, Xiang H, Wang B, Cao Y (2016) Genetic analysis of DACT1 in 100 Chinese Han women with Mullerian duct anomalies. Reprod Biomed Online 32:420–426. https://doi.org/10.1016/j.rbmo.2016.01.003
